# C1ql1-Bai3 signaling is necessary for climbing fiber synapse formation in mature Purkinje cells in coordination with neuronal activity

**DOI:** 10.1186/s13041-023-01048-4

**Published:** 2023-07-24

**Authors:** Takahiro Aimi, Keiko Matsuda, Michisuke Yuzaki

**Affiliations:** Department of Physiology, Keio University School of Medicine, Tokyo, 160-8582 Japan

**Keywords:** Cerebellum, Purkinje cell, Climbing fiber, Synapse, Electrophysiology, C1ql1, Bai3

## Abstract

Changes in neural activity induced by learning and novel environments have been reported to lead to the formation of new synapses in the adult brain. However, the underlying molecular mechanism is not well understood. Here, we show that Purkinje cells (PCs), which have established adult-type monosynaptic innervation by climbing fibers (CFs) after elimination of weak CFs during development, can be reinnervated by multiple CFs by increased expression of the synaptic organizer C1ql1 in CFs or Bai3, a receptor for C1ql1, in PCs. In the adult cerebellum, CFs are known to have transverse branches that run in a mediolateral direction without forming synapses with PCs. Electrophysiological, Ca^2+^-imaging and immunohistochemical studies showed that overexpression of C1ql1 or Bai3 caused these CF transverse branches to elongate and synapse on the distal dendrites of mature PCs. Mature PCs were also reinnervated by multiple CFs when the glutamate receptor GluD2, which is essential for the maintenance of synapses between granule cells and PCs, was deleted. Interestingly, the effect of GluD2 knockout was not observed in Bai3 knockout PCs. In addition, C1ql1 levels were significantly upregulated in CFs of GluD2 knockout mice, suggesting that endogenous, not overexpressed, C1ql1-Bai3 signaling could regulate the reinnervation of mature PCs by CFs. Furthermore, the effects of C1ql1 and Bai3 overexpression required neuronal activity in the PC and CF, respectively. C1ql1 immunoreactivity at CF-PC synapses was reduced when the neuronal activity of CFs was suppressed. These results suggest that C1ql1-Bai3 signaling may mediate CF synaptogenesis in mature PCs, potentially in concert with neuronal activity.

**Supplementary Information:**

The online version contains supplementary material available at 10.1186/s13041-023-01048-4.

## Introduction

Neural circuits are formed and refined in response to changes in neural activity associated with learning and new environments, not only during development but throughout life [1–4]. The increasing evidence suggests that such structural plasticity plays a key role in the pathophysiology of various neurological and neuropsychiatric disorders [5, 6]. However, the molecular mechanisms underlying structural plasticity in adult brains remain largely unknown, partly due to the complexity of neuronal circuits consisting of many heterogenous synapses. Activity-dependent structural plasticity during development has been extensively studied in the cerebellar cortex, which contains simple and well-defined neuronal circuits. Purkinje cells (PCs), which send the only outputs from the cerebellar cortex, receive two excitatory inputs, parallel fibers (PFs) from granule cells and climbing fibers (CFs) from the inferior olive neurons (IONs) at proximal and distal dendrites, respectively. Several hundred thousand PFs make synapses on spines located on the distal dendrites of a single PC. In contrast, although multiple CFs initially innervate a single immature PC, a single CF becomes dominant in an activity-dependent manner, forming synapses on the proximal dendrites while the rest of the CFs are eliminated by the end of the 3rd postnatal week in mice [7, 8]. Interestingly, the cerebellar cortex undergoes remarkable changes in its architecture after damage or alteration of neural activity, even in adulthood. For example, PC spine density is increased by motor-skill learning [9] or exposure to an enriched environment [10]. Conversely, inhibition of neuronal activities by application of the Na^+^ channel blocker tetrodotoxin or the AMPA-receptor antagonist NBQX to the adult cerebellar cortex replaced CF synapses with PF synapses on proximal dendrites [11–13]. Thus, the cerebellar cortical circuits provide a valuable model to elucidate molecular mechanisms underlying structural plasticity in the adult brain.

Many synaptic organizers regulate synapse formation, maturation, or elimination during development [14]. Among them, the C1q family is crucial in organizing excitatory inputs to PCs during development [15]. Cbln1, a member of the C1q family secreted from PFs, regulates PF-PC synapses by binding to the GluD2 glutamate receptor expressed on distal dendrites of PCs [16, 17]. In contrast, C1ql1, provided by CFs, mediates CF-PC synapse formation on the proximal dendrites by binding to the adhesion G-protein-coupled receptor Bai3 [18, 19]. Interestingly, both Cbln1-GluD2 and C1ql1-Bai3 protein pairs remain expressed in the adult cerebellum after PF- and CF-PC synapses are established. When C1ql1 or Bai3 is deleted in the IONs or PCs, respectively, CF synapses are lost from the proximal dendrites of PCs in the adult cerebellum [18]. Similarly, when GluD2 is deleted in the mature PCs, PF synapses are gradually lost from the distal dendrites [20]. Furthermore, although PCs are normally innervated by single CFs on proximal dendrites, the loss of GluD2 in the adult cerebellum causes multiple CFs to innervate distal PC dendrites where PFs normally make synapses [20]. Intriguingly, similar changes in the innervation pattern of PCs by PFs and CFs are observed when neuronal activity is inhibited in the adult cerebellum [11–13]. Furthermore, Cbln1 is secreted from PFs in an activity-dependent manner [21], while Cbln1 mRNA expression is repressed by prolonged neuronal activities [22, 23]. These results suggest that C1q family proteins may play a role in activity-dependent structural plasticity in the cerebellum after PF- and CF synapses are matured.

In the present study, we investigated whether and how C1ql1-Bai3 signaling is involved in CF-PC structural plasticity in mature cerebellar circuits. We found that when the expression of C1ql1 and Bai3 was upregulated in CFs and PCs, respectively, transverse branches of CFs elongated and formed new synapses with distal dendrites, allowing PCs to be re-innervated by multiple CFs. This process required neural activity of both CFs and PCs, suggesting that C1ql1-Bai3 signaling may be involved in the activity-dependent CF synapse modification in the mature PCs.

## Methods

### Animals

All procedures relating to the care and treatment of mice were performed in accordance with the guidelines approved by the animal resource committee of Keio University. Mice of the following strains were used: *Htr5B-tTA* and *tetO-YC* mice [25] (a gift from Prof. Kenji Tanaka, Keio Univ Sch Med), conditional *Bai3* knockout mice (*Bai3*^*f/f*^) [18], conditional GluD2 (*Grid*2^*f/f*^) mice [20], GluD2 knockout mice [17], C57B6/N mice (Japan SLC, Inc.). The constitutive *Bai3* knockout mouse was generated by crossing *Bai3*^*f/f*^ with telencephalin-*Cre* transgenic mice as previously reported [30, 37]. Mice were housed with a 12:12 h light-dark cycle with food and water available ad libitum. The sex for the virus injections, immunohistochemistry, and electrophysiology was not distinguished.

### Cell lines

HEK293 cells (tSA line; gift from Dr. R. Horn, Thomas Jefferson Univ., PA) and AAV-293 cells (Cell Biolab) were cultured with Dulbecco’s modified Eagle medium (DMEM, D5796, Sigma-Aldrich) with 10% Fetal Bovine Serum (#004–00025, Japan Bioserum), 50 U/mL penicillin/streptomycin (15140-122, Thermo Fisher Scientific), and 2 mM L-Glutamine. Cells were incubated at 37 °C in the 10% CO_2_ incubator.

### cDNA constructs

Some constructs of Bai3, C1ql1 and plasmids for lentivirus preparation were previously reported [18]. Bai3 mutants were generated by two-step overlapping PCRs. To visually detect the cells expressing genes of interest, the fluorescent proteins (GFP, YFP or mCherry) are linked by a self-cleaving 2 A peptide (P2A, F2A) from the foot-and-mouth-disease virus were co-expressed [18]. cDNAs encoding ChR2-eYFP (#110,339), tdTomato (#104,112) and Cre (#107,738) were obtained from Addgene. The minimal L7 promoter [39] was synthesized (Eurofin genomics). The sequence of ESKir2.1 and pAAV-DJ was kindly provided by Dr. Kazuya Togashi (Grad School of Science, Univ. Tokyo). pAAV-TRE, pAAV-L7 and pAAV-Syn-DIO vectors were purchased from Addgene (#104,112, #58,867 and #50,459). pHelper were purchased from Cell Biolab.

### Virus preparation

Lentiviruses were used to express Bai3 or Cre in PCs. Lentivirus was prepared as previously reported [18] with small modifications. The cDNAs were cloned into the pCL20c vector and expressed under the MSCV promoter. Lentiviruses were produced by transfecting the pCL20c vector and three helper plasmids (pCAG-KGRIR, pCAG-RTR2 and pCAG-VSVG) into HEK293tSA cells using a calcium phosphate method. 36–40 h after the transfection, the culture media were collected and centrifuged at 24,000 rpm for 2 h at 2 ℃ to concentrate the virus. After the centrifugation, the pellets were dissolved with a small volume of cold medium and stored frozen at -80 ℃. The lentivirus was at a 10^8–9^ titer unit.

AAV-DJ vectors were used to express C1ql1 in CFs and ESKir2.1 and Cre in PCs. AAVs were produced by transfecting the pAAV vector, pHelper and pAAV-DJ into 293AAV (Cell Biolab, lnc) using a calcium phosphate method. 36–40 h after transfection, cells were collected and AAVs were purified by using an AAV purification kit (Takara #6666) following the manufacturer’s protocols. As for the pAAV vectors, SmaI digestion was performed to confirm that the two SmaI sites within the ITR were maintained. The titer of AAVs was determined by using the AAVpro Titration Kit (Takara #6233) and calculated as 10^10–11^ vector genomes/mL.

### Stereotaxic injection

Mice (3–4 weeks) were anesthetized with a mixture of ketamine (80 mg/kg body weight) and xylazine (20 mg/kg body weight) by intraperitoneal injection. To express the genes into PC, glass pipettes filled with the AAV and/or lentivirus solution (2–4 µL) were inserted into the cerebellar vermis at the depth of 0.5 mm. The solution was injected at a speed of 0.25 µL/min.

To express genes in the CFs, glass pipettes were inserted into the ION according to the reported method with small modifications [40]. The AAV solution (1.0–1.5 µL) was injected at a speed of 0.2 µL/min. After the injections, the incised skin was closed by using adhesive and the mice transiently stayed in the recovery cages for 12–16 h. Then mice were returned to their home cages.

### Electrophysiology

Mice were anesthetized with isoflurane and the brains were rapidly removed and immersed in an ice-cold choline-based cutting solution containing (in mM): 120 Choline Cl, 3 KCl, 1.25 NaH_2_PO_4_, 28 NaHCO_3_, 8 MgCl_2_, 22 Glucose and 0.5 Ascorbate, bubbled with a mixture of 95%O_2_ and 5%CO_2_. Parasagittal cerebellar slices (200-µm thick) were prepared by using a micro-slicer (Pro7N, Dosaka EM) in the ice-cold cutting solution. The prepared slices were transferred into the artificial cerebrospinal fluid (ACSF) containing (in mM) 125 NaCl, 2.5 KCl, 2 CaCl_2_, 1 MgCl_2_, 26 NaH_2_PO_4_, 10 Glucose and 0.1 picrotoxin, bubbled with a mixture of 95%O_2_ and 5%CO_2_. The transferred slices were incubated with ACSF at room temperature for more than 1 h to recover.

Whole-cell patch-clamp recordings were made from visually identified PCs using an x60 water-immersion objective attached to an upright microscope (BX51WI, Olympus) at room temperature. Intracellular solutions were composed of (in mM): 150 Cs-gluconate, 10 HEPES, 4 MgCl_2_, 4 Na_2_ATP, 1 Na_2_GTP, 0.4 EGTA and 5 lidocaine N-ethyl bromide (QX-314) (pH 7.25, 290–300 mOsm/kg) for the CF- and PF-EPSC recordings. The patch pipette resistance was 1–2 MΩ.

To evoke CF-EPSCs, a glass pipette filled with ACSF was placed on the granular layer near (20–100 μm) the PC voltage-clamped at -10 mV and square paired pulses (20 µs duration, 0–300 µA and 50 ms inter-stimulus interval) were applied. Selective stimulation of CFs was confirmed by the paired-pulse depression of EPSC amplitudes. The number of functional CF synapses on single PCs was estimated by varying the stimulus intensity because a single CF input has a single threshold for excitation.

To estimate the location of stimulation for evoking main or surplus CF-EPSC, the XY coordinates of the stimulation location were defined with the center of the PC soma as the origin. The stimulus electrode was moved systematically every 10 μm in the XY direction on the surface of the granular layer. Square paired pulses (20 µs duration, 0–300 µA and 50 ms inter-stimulus interval) were applied at about 40 stimulation points, respectively. Nearest distances for evoking main or surplus CF-EPSC were calculated as the mean of 3 stimulation points closest to the origin, at which main or surplus CF-EPSC were evoked.

To ensure that EPSCs were evoked by CFs transduced by AAV, we optogenetically stimulated CFs by co-expressing ChR2 in IO neurons in Figs. 2 and 7. Light stimulation (wavelengths: ~470 nm, 4–10 ms duration, 100 ms inter-stimulus interval) was applied from a mercury lamp (Olympus) combined with a mechanical shutter. The number of functional CF synapses on single PCs was estimated by varying the light intensities (0–4 mW/mm^2^) and durations. Some PCs (15–20%) did not respond to light stimulation, most likely because not all CFs expressed ChR2. Due to the exclusion of non-responding PCs, the number of functional CFs determined by optogenetic CF stimulation is likely an underestimation. Indeed, unlike electric stimulation of CFs [18], optogenetic stimulation failed to detect multiple CF inputs on single PCs in Bai3 knockout mice (Fig. 2H).

To evoke PF-EPSCs, square paired pulses (20 µs duration, 0–200 µA and 50 ms inter-stimulus interval) were applied through a glass pipette placed on the molecular layer. PCs were voltage-clamped at -80 mV. Selective stimulation of PFs was confirmed by the paired-pulse potentiation of EPSC amplitudes.

For recording mIPSCs, intracellular solutions were composed of (in mM): 120 Cs-Chloride, 20 HEPES, 1 MgCl_2_, 4 Na_2_ATP, 10 sucrose (pH 7.25, 290 mOsm/kg). mIPSC were recorded from PCs in ACSF containing (µM): 10 NBQX, 20 D-AP5 and 1 TTX, in which picrotoxin was not added. PCs were voltage-clamped at -70 mV and mIPSCs were recorded as inward currents.

Current responses were recorded with an Axopatch 200B amplifier (Molecular Devices) and pClamp software (version 10, Molecular Devices) was used for data acquisition and analysis. Signals were filtered at 1 kHz and digitized at 4 kHz.

### Immunohistochemistry

Mice were anesthetized by intraperitoneal injection of 2% avertin and fixed by perfusion with 4%PFA/0.1 M sodium phosphate buffer (PB). Fixed brains were submerged in 4%PFA/0.1 M PB at 4℃ for 12–16 h and solutions were replaced with PBS containing 0.1% sodium azide. Brains were embedded in a 2% agarose gel just before sectioning. The cerebellar cortex (50 μm thickness, sagittal or coronal) and the brainstem, including the IONs (50 μm, coronal), were cut by a micro-slicer (DTK-1000; Dosaka EM).

Immunostaining was performed in glass tubes. After sections were treated with 10% donkey serum, primary antibodies (Guinea pig anti-GFP [Frontier Institute], Goat anti-GFP [Frontier Science], Chicken anti-GFP [Millipore], Rat anti-mCherry [Thermo Fisher Scientific], Rabbit anti-vGluT2 [Frontier Institute], Guinea pig anti-vGluT2 [Frontier Institute], Goat anti-vGluT2 [Frontier Institute], Guinea pig anti-C1ql1 [a gift from Masahiko Watanabe], Goat anti-calbindin[Frontier Institute], Rabbit anti-Bai3 [a gift from Masahiko Watanabe], Rabbit anti-HA [Cell Signaling Technology], Rabbit anti-c-Fos [Merck]) were applied and incubated at room temperature for 12–16 h. The specificity of the antibodies against C1ql1 and Bai3 was previously confirmed by the lack of immunoreactivity in *C1ql1* and *Bai3* knockout mice, respectively [18]. Sections were washed with PBS 3 times and incubated with secondary antibodies, which were conjugated with fluorescence dye such as Dylight 405, Alexa 488, 594, 647 and Cy3 (Molecular probes or Jackson ImmunoResearch Laboratories) against the respective primary antibody, together with DAPI at room temperature for 2 h. Sections were washed with PBS and mounted on slide glasses with fluoromount G (Invitrogen). For staining HA-C1ql1, pepsin treatment (37℃ for 5 min) was performed to expose antigens before treatment with 10% donkey serum. Fluorescent images were taken by using confocal microscopy (FV1000, Olympus). To observe and trace transverse CF branches, z-stack images (1 μm step for 30 μm) were taken. Images were analyzed by using Fiji software. Regions of interest (ROI), such as cell soma and vGluT2 puncta, were selected manually, and signal intensities were measured in each ROI. Transverse CF branches were manually traced, and the length of each branch was measured

### Ca^2+^ imaging

Ca^2+^ imaging was performed in PCs under the current clamp condition using confocal laser-scanning microscopy (FV-1200, Olympus). Intracellular solutions were composed of (in mM): 130 K-Gluconate, 10 KCl, 10 HEPES, 1 MgCl_2_, 4 Na_2_ATP, 1 Na_2_GTP, 15 sucrose and 0.1 Oregon Green 488 BAPTA-1 (OGB-1) (pH 7.25, 315 mOsm/kg). We waited at least 20 min after establishing the whole-cell mode to fill PC dendrites with OGB-1 and identified the stimulation site for evoking main and surplus CF inputs by CF-EPSC recording. Fluorescence images were acquired at 5–10 Hz while recording CF-evoked voltage changes. In some cases, the extracellular Ca^2+^ concentration was increased from 2 mM to 5 mM to enhance Ca^2+^ changes induced by surplus CF stimulations.

The acquired fluorescence images were analyzed by using Fiji software. CF-evoked Ca^2+^ changes were expressed as increases in the fluorescence value (ΔF) divided by the averaged fluorescence value before CF stimulations (F_0_). The area that showed a large Ca elevation (ΔF/F_0_ within 30% of the peak value) was analyzed. Voltage responses were recorded with an Axopatch 200B amplifier (Molecular Devices) and pClamp software (version 10, Molecular Devices) was used for data acquisition. Signals were filtered at 1 kHz and digitized at 4 kHz for the evoked voltage changes.

### Statistical analyses

**Fig. 1:**
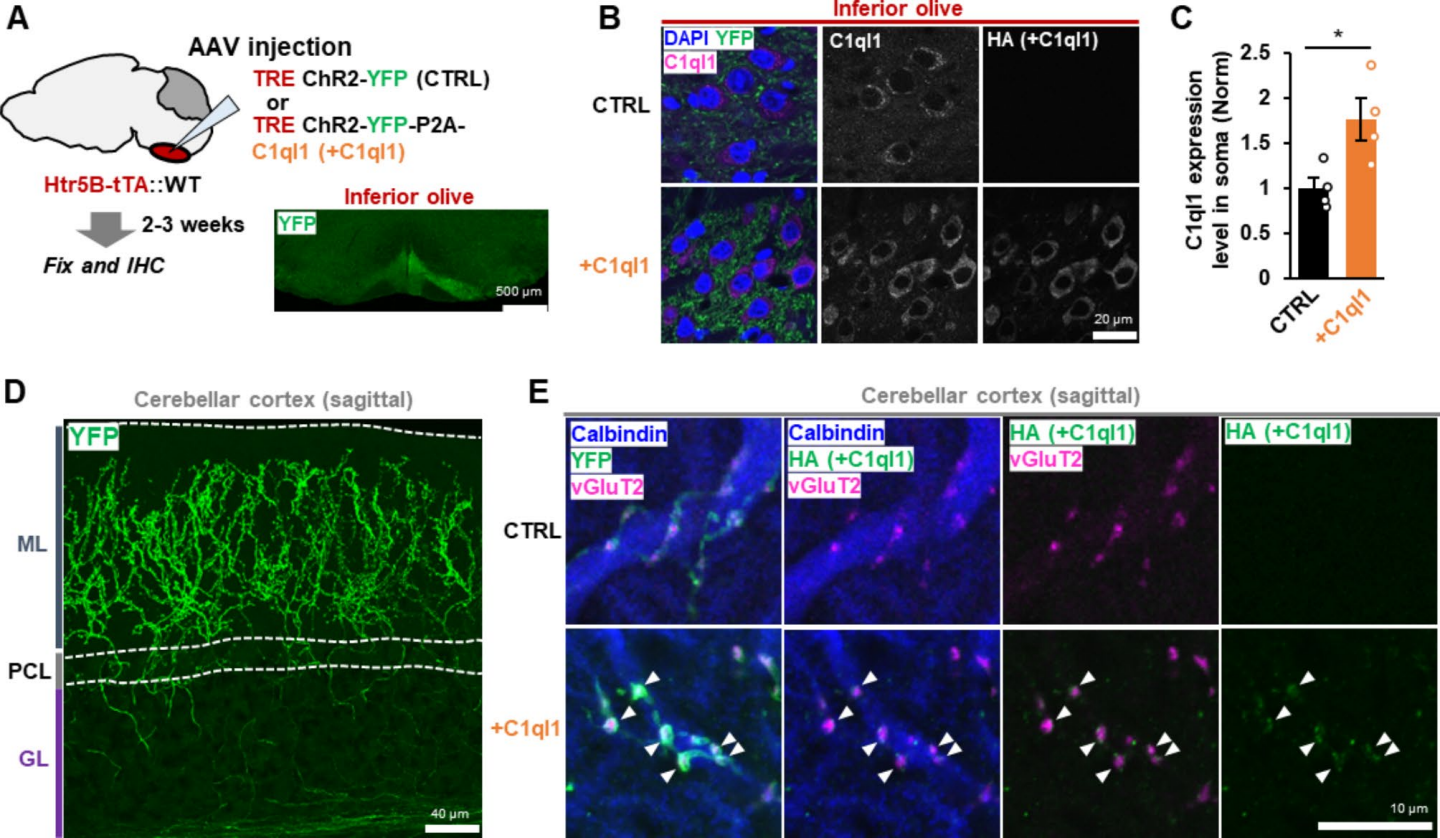
CF selective gene delivery using Htr5B-tTA mice and AAV **A** Experimental scheme. AAVs encoding ChR2-YFP with or without C1ql1 were injected into the ION. The right panel shows YFP signals in the ION. Scale bar, 500 μm. **B** Expression levels of C1ql1 in the ION. Immunohistochemical staining shows total C1ql1, exogenous HA-C1ql1 and YFP in the ION infected with AAV-CTRL and AAV-C1ql1. DAPI staining shows the nucleus. Scale bar, 20 μm. **C** Quantification of C1ql1 immunoreactivity in the soma of IONs (**B**_**2**_). p = 0.0389, Two-tailed Welch’s t-test; n = 4 mice each. **D** Selective expression of YFP and C1ql1 in the CFs. No YFP signals were detected in the mossy fibers in the granular layer. ML, molecular layer; PCL, Purkinje cell layer; GL, granular layer. Scale bar, 40 μm. **E** Immunohistochemical staining of vGluT2 (magenta), YFP or HA-C1ql1 (green) and calbindin (blue) indicates accumulation of HA-C1ql1 at CF synapses. Scale bar, 10 μm. Bars represent mean ± SEM. *p < 0.05

**Fig. 2:**
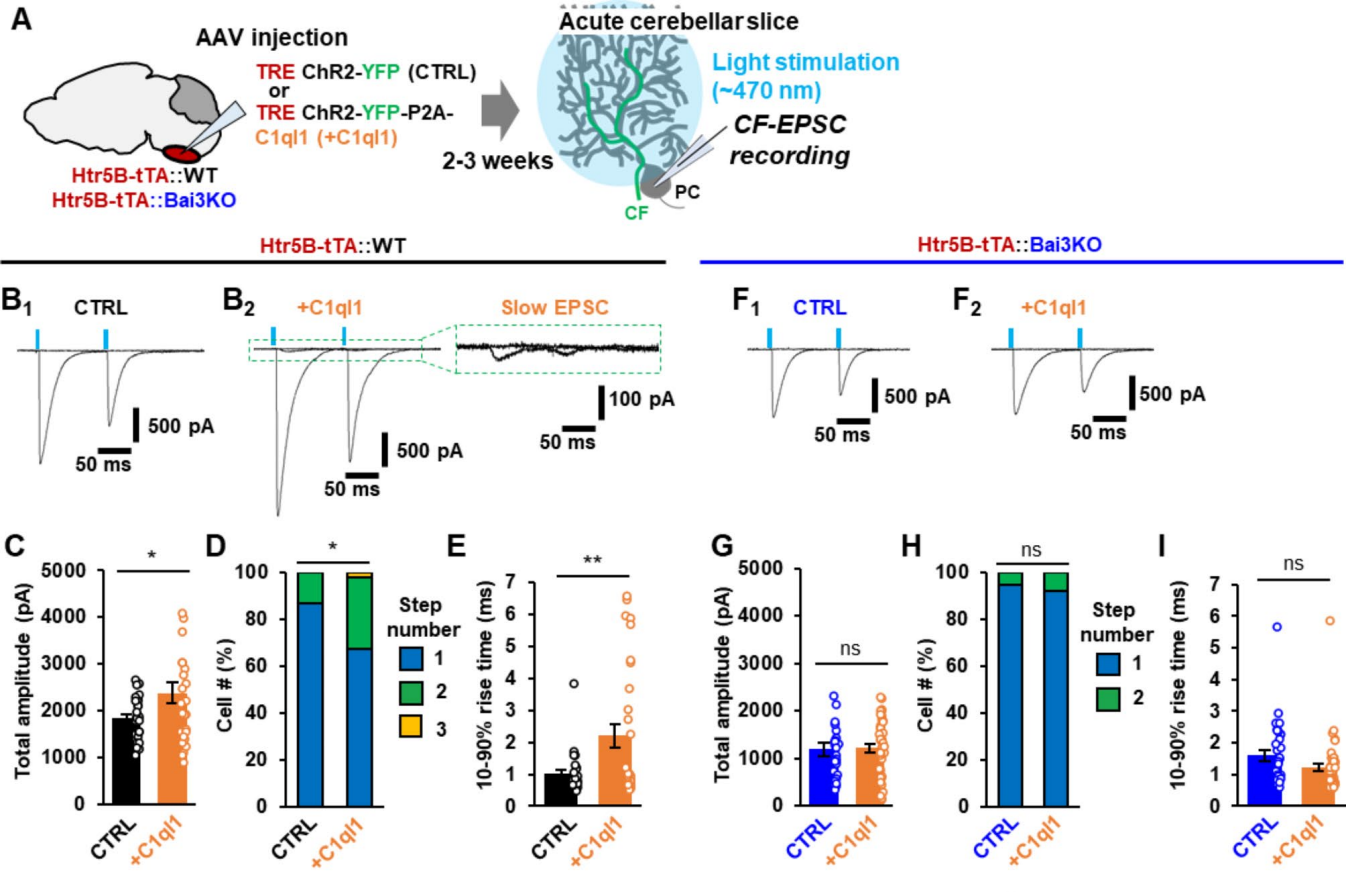
Increased C1ql1 levels in CFs allow adult PCs to be innervated by multiple CFs **A** Experimental scheme for recording CF-EPSCs. **B** Representative CF-EPSC traces from wild-type PCs. The blue bar indicates the timing of light stimulation. Increasing the light intensity elicited a single EPSC in control slices (**B**_**1**_) in an all-or-none manner, but multiple EPSCs with a slower rise time in slices overexpressing C1ql1 (**B**_**2**_). **C** Total CF-EPSC amplitude. The graph shows the sum of the peak amplitudes of single CF-EPSCs or multiple CF-EPSCs. p = 0.0372, two-tailed Welch’s t-test; n = 28 cells from 4 mice (CTRL); n = 29 cells from 5 mice (+ C1ql1). **D** The percentage of the number of CFs innervating single PCs. The number of EPSCs evoked by distinct CF activation thresholds (number of steps) is shown. p = 0.0234, Mann–Whitney U test; n = 51 cells from 4 mice (CTRL); n = 49 cells from 5 mice (+ C1ql1). **E** Average of the 10–90% rise time of CF-EPSCs. p = 0.0028, two-tailed Welch’s t-test; n = 28 responses from 4 mice (CTRL); n = 46 responses from 5 mice (+ C1ql1). **F** Representative CF-EPSC traces from Bai3 knockout PCs (left: CTRL, right: +C1ql1). **G** Total CF-EPSC amplitude. The graph shows the sum of peak amplitudes of single CF-EPSCs or multiple CF-EPSCs. p = 0.8534, two-tailed Welch’s t-test; n = 31 cells from 3 mice (CTRL); n = 42 cells from 4 mice (+ C1ql1). **H** The percentage of the number of CFs innervating single PCs. p = 0.6159, Mann–Whitney U test; n = 38 cells from 3 mice (CTRL); n = 50 cells from 4 mice (+ C1ql1). **I** Average of the 10–90% rise time of CF-EPSCs. p = 0.0609, two-tailed Welch’s t-test; n = 33 responses from 3 mice (CTRL); n = 46 responses from 4 mice (+ C1ql1). Bars represent mean ± SEM. **p < 0.01; *p < 0.05; ns, not significant

**Fig. 3:**
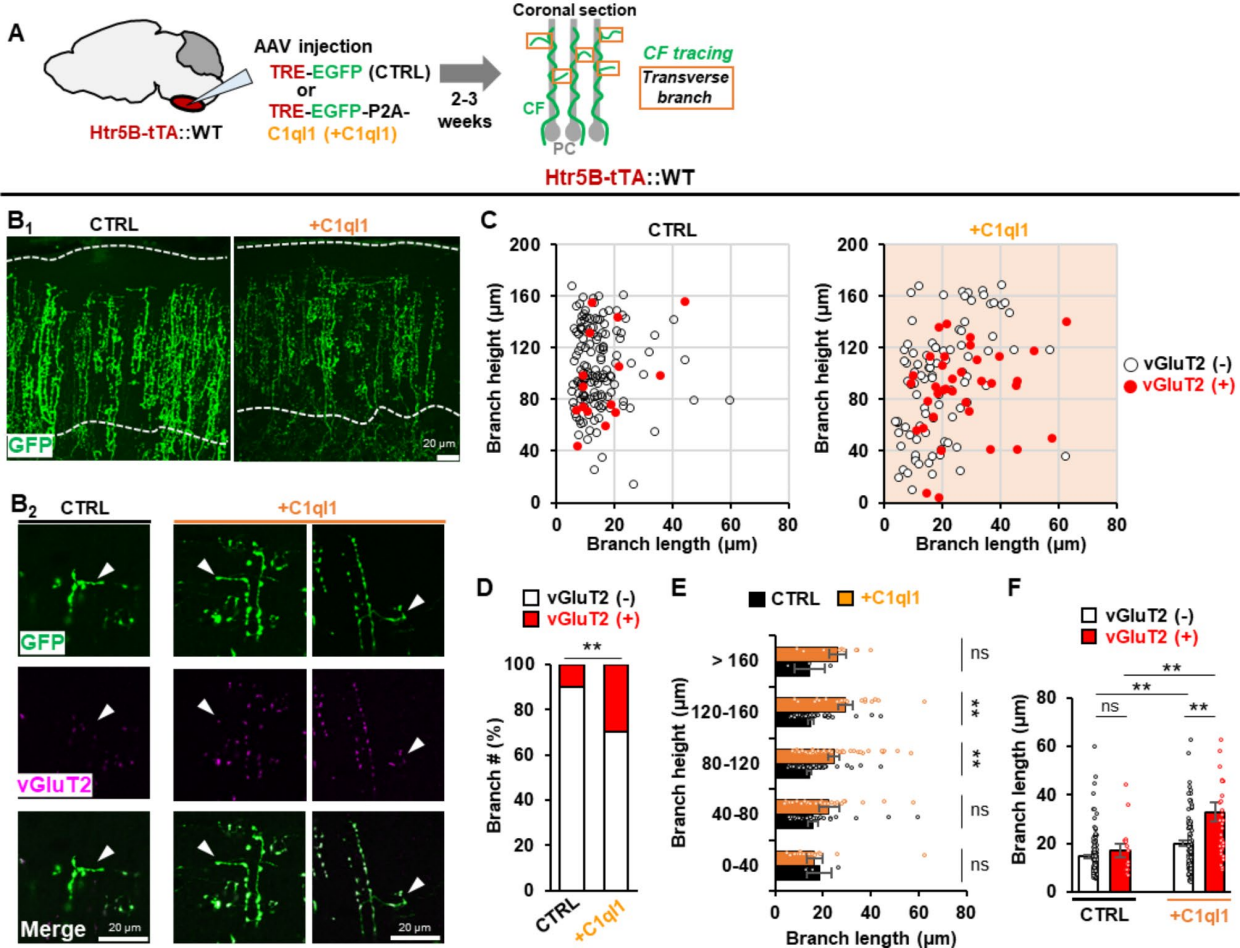
C1ql1-Bai3 signaling induces synapse formation by transverse CF branches **A** Experimental scheme. **B** Coronal cerebellar sections. GFP (CTRL) or GFP plus C1ql1 (+ C1ql1) was overexpressed in CFs. Maximum intensity z-projection images are shown. Dotted lines, upper and lower boundaries of the molecular layer (**B**_**1**_). Enlarged views of representative CF branches (**B**_**2**_). Arrowheads indicate vGluT2-negative and positive branches in CTRL and + C1ql1, respectively. Scale bars, 20 μm. **C** Height and length of CF transverse branches in the molecular layer. Branch height was measured from the apical pole of PC somata. Transverse branches negative (-) and positive (+) for vGluT2 are indicated by white and red circles, respectively. n = 152 branches (CTRL), n = 132 branches (+ C1ql1). **D** Percentage of vGluT2-positive CF transverse branches in CTRL or + C1ql1. p = 1.352 × 10^− 5^, n = 152 (CTRL); n = 132 (+ C1ql1). Mann–Whitney U test. **E** Histogram showing the mean length of CF transverse branches as a function of their height in the molecular layer. Black and orange bars represent the cerebellum in CTRL and + C1ql1, respectively. 0–40 μm: p = 0.8328, n = 3 (CTRL), n = 18 (+ C1ql1); 40–80 μm: p = 0.1731, n = 34 (CTRL), n = 36 (+ C1ql1); 80–120 μm: p = 2.648 × 10^− 5^, n = 63 branches (CTRL), n = 58 (+ C1ql1); 120–160 μm: p = 7.370 × 10^− 5^, n = 49 (CTRL), n = 20 (+ C1ql1); >160 μm: p = 0.1321, n = 3 (CTRL), n = 9 (+ C1ql1). Two-tailed Welch’s t-test. **F** Histogram showing the mean length of CF transverse branches with the presence or absence of vGluT2. CTRL: p = 0.4338; vGluT2(-), n = 137; vGluT2(+), n = 15. +C1ql1: p = 0.003482; vGluT2(-), n = 93; vGluT2(+), n = 39. Two-tailed Welch’s t-test. All datasets are from 3 mice per group (CTRL and + C1ql1). Bars represent mean ± SEM. **p < 0.01; *p < 0.05; ns, not significant

**Fig. 4:**
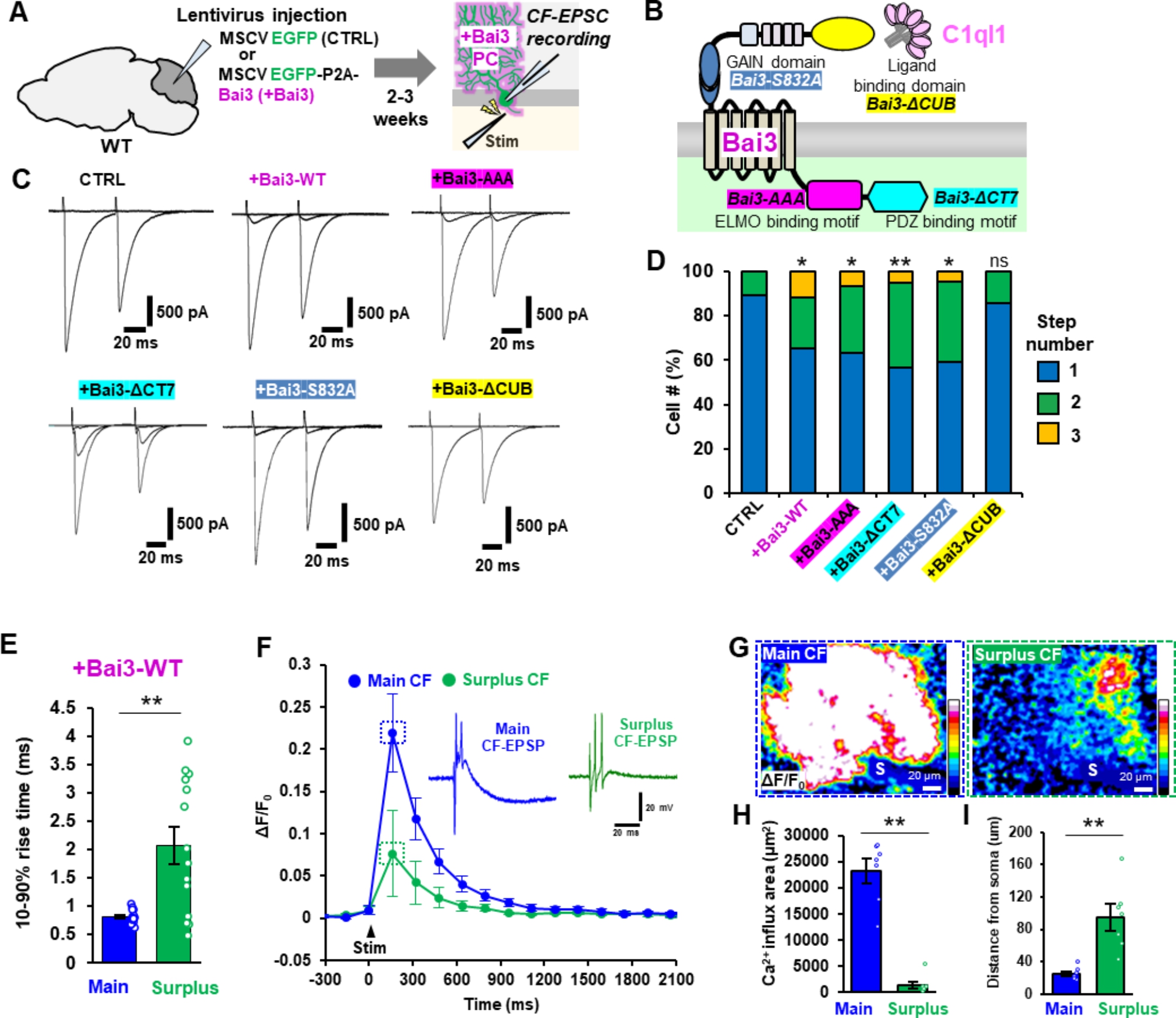
Bai3 overexpression in PCs induces re-innervation by CFs at distal dendrites by binding to C1ql1 **A** Experimental scheme. **B** Diagram of the functional domains of Bai3 and its mutants. **C** Representative CF-EPSC traces recorded from adult wild-type PCs overexpressing the indicated constructs. Paired-pulse stimulation with 50-ms interstimulus interval was applied. **D** The percentages of the number of CFs innervating single PCs. The number of EPSCs evoked by distinct CF activation thresholds (step numbers) is shown. Bai3-WT: p = 0.0100, n = 69 cells from 11 mice; Bai3-AAA: p = 0.0278, n = 30 cells from 4 mice; Bai3-ΔCT7: p = 0.0007, n = 60 cells from 8 mice; Bai3-S832A: p = 0.0186, n = 22 cells from 3 mice; Bai3-ΔCUB: p = 0.9981, n = 63 cells from 8 mice. Kruskal-Wallis test followed by Steel test vs. CTRL: n = 57 cells from 7 mice. **E** Average of the 10–90% rise time of CF-EPSCs in PCs overexpressing Bai3-WT. p = 0.0018, Two-tailed Welch’s t-test, n = 24 traces (main), n = 14 traces (surplus) from 11 mice. **F** Time course of CF-evoked Ca^2+^ changes associated with main and surplus EPSPs. Changes in the fluorescence (ΔF) were normalized by the averaged fluorescence (F_0_) before the CF stimulation (arrowhead). Inset, representative CF-EPSPs during Ca^2+^ imaging. **G** Representative CF-evoked Ca^2+^ changes associated with main and surplus EPSPs. S, PC soma. Scale bar, 20 μm. **H** The mean area of Ca^2+^ elevation associated with main or surplus CF-EPSPs. The area of large Ca^2+^ elevation (ΔF/F_0_ within 30% of the peak value) was measured. p = 2.763 × 10^− 5^, two-tailed Welch’s t-test, n = 7 responses (main), n = 7 (surplus) from 5 mice. **I** The closest distance between the site of large Ca^2+^ elevation and the PC soma was measured (see Methods). p = 0.0075, two-tailed Welch’s t-test, n = 7 responses (main), n = 7 (surplus) from 5 mice. Bars represent mean ± SEM. **p < 0.01; *p < 0.05; ns, not significant

**Fig. 5:**
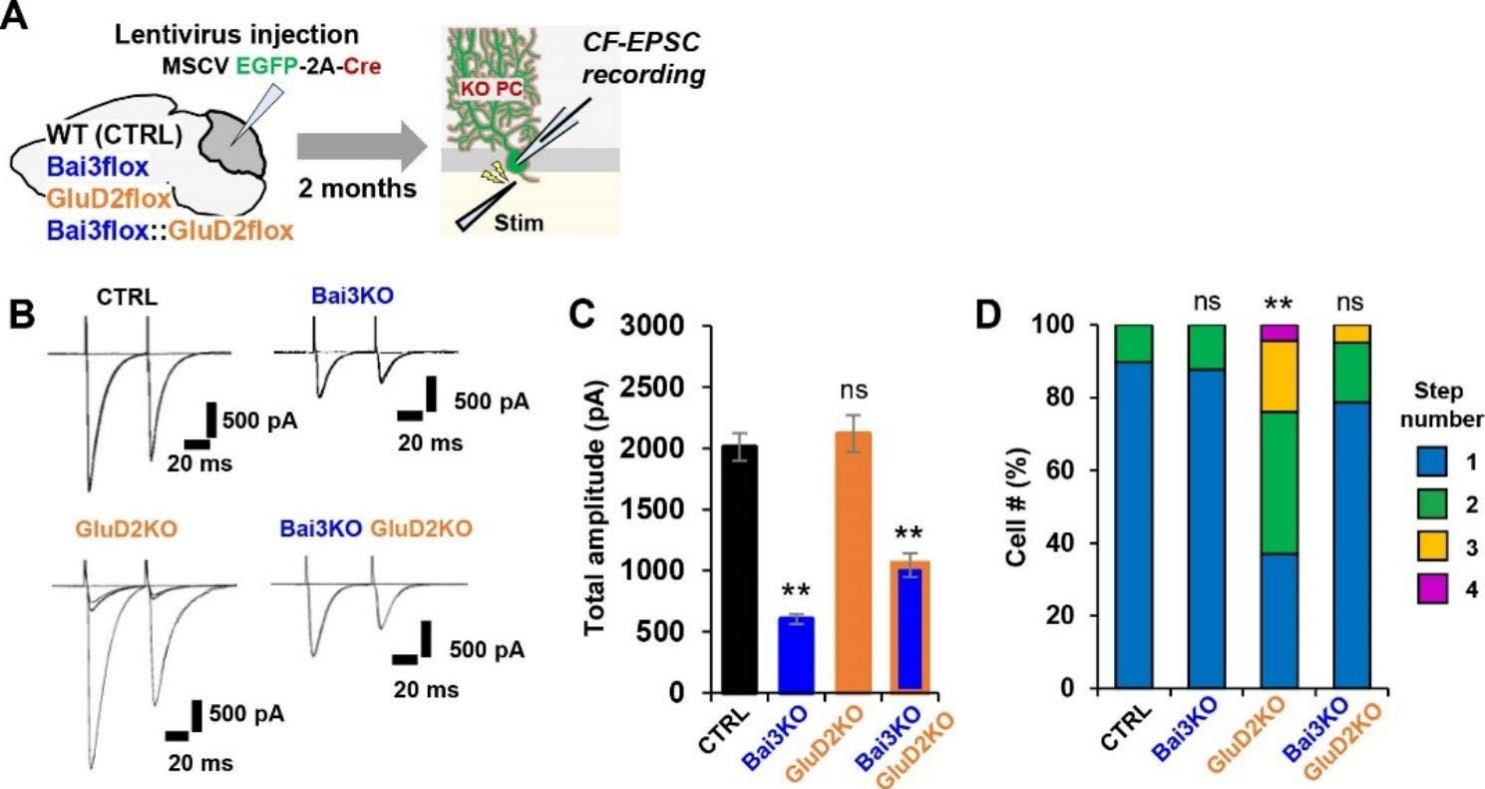
Endogenous Bai3 is required for synapse formation of re-innervating CFs **A** Experimental scheme. **B** Representative CF-EPSC traces recorded from PCs of the indicated genotypes. **C** Total CF-EPSC amplitude. The graph shows the sum of peak amplitudes of single CF-EPSCs or multiple CF-EPSCs. CTRL (n = 17 cells from 2 mice) vs. Bai3 knockout (KO), p = 1.272 × 10^− 6^ (n = 29 cells from 5 mice); vs. GluD2 KO, p = 0.8311 (n = 25 cells from 5 mice); vs. Bai3 KO::GluD2 KO, p = 1.336 × 10^− 6^ (n = 33 cells from 4 mice). One way ANOVA followed by Dunnett’s test. **D** The percentages of the number of CFs innervating single PCs. The number of EPSCs evoked by distinct CF activation thresholds (step numbers) is shown. CTRL (n = 29 cells from 2 mice) vs. Bai3 KO, p = 0.9859 (n = 49 cells from 5 mice); vs. GluD2 KO, p = 1.885 × 10^− 5^ (n = 46 cells from 5 mice); vs. Bai3 KO::GluD2 KO, p = 0.3750 (n = 61 cells from 4 mice). Kruskal-Wallis test followed by Steel test. Bars represent mean ± SEM. **p < 0.01; ns, not significant

**Fig. 6:**
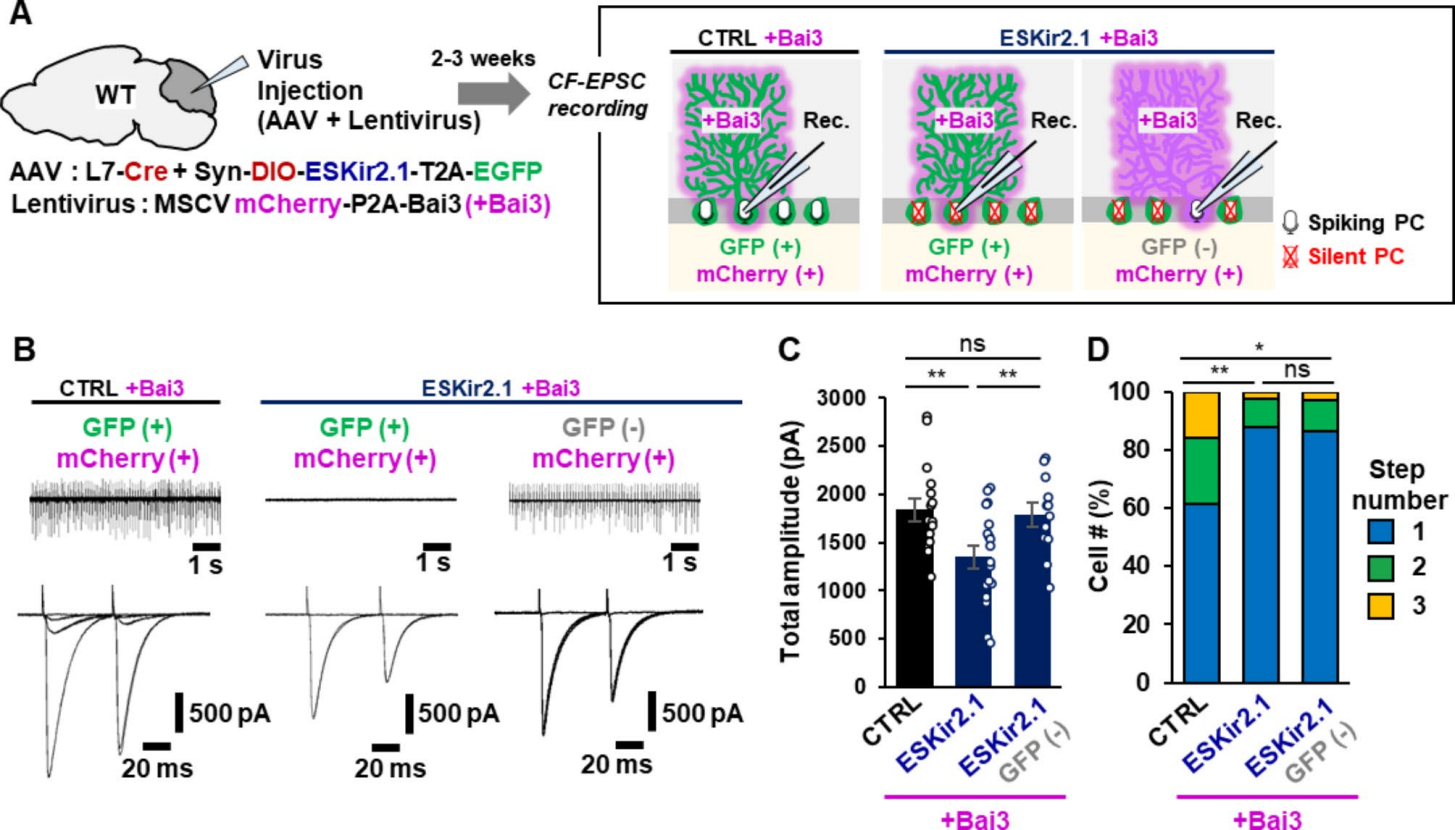
Bai3-induced re-innervation by CFs requires PC activity **A** Experimental scheme. The right panel shows three cases: in all cases recorded PCs overexpress Bai3, but all PCs are active (spiking PCs, left), all PCs are silenced by ESKir2.1 (silent PCs, middle) and recorded PCs are active but neighboring PCs are silenced (right). **B** Representative action potentials by loose-patch recordings (upper traces) and CF-EPSCs by whole-cell patch-clamp recordings (lower traces) from PCs expressing the indicated constructs. **C** Total CF-EPSC amplitude. The graph shows the sum of peak amplitudes of single CF-EPSCs or multiple CF-EPSCs. p = 0.0098, CTRL + Bai3 vs. ESKir2.1 + Bai3; p = 0.9500, CTRL + Bai3 vs. ESKir2.1 + Bai3, GFP (-); p = 0.0393, ESKir2.1 + Bai3 vs. ESKir2.1 + Bai3, GFP (-). One way ANOVA followed by Tukey’s test. n = 16 cells from 3 mice (CTRL + Bai3), n = 18 cells from 3 mice (ESKir2.1 + Bai3), n = 12 cells from 3 mice (ESKir2.1 + Bai3, GFP (-)). **D** The percentages of the number of CFs innervating single PCs. The number of EPSCs evoked by distinct CF activation thresholds (step numbers) is shown. p = 0.0061, CTRL + Bai3 vs. ESKir2.1 + Bai3; p = 0.0182, CTRL + Bai3 vs. ESKir2.1 + Bai3, GFP (-); p = 0.9894, ESKir2.1 + Bai3 vs. ESKir2.1 + Bai3, GFP (-). Kruskal-Wallis test followed by Steel test n = 44 cells from 5 mice (CTRL + Bai3), n = 49 cells from 9 mice (ESKir2.1 + Bai3), n = 37 cells from 6 mice (ESKir2.1 + Bai3, GFP (-)). Bars represent mean ± SEM. **p < 0.01; *p < 0.05; ns, not significant

**Fig. 7:**
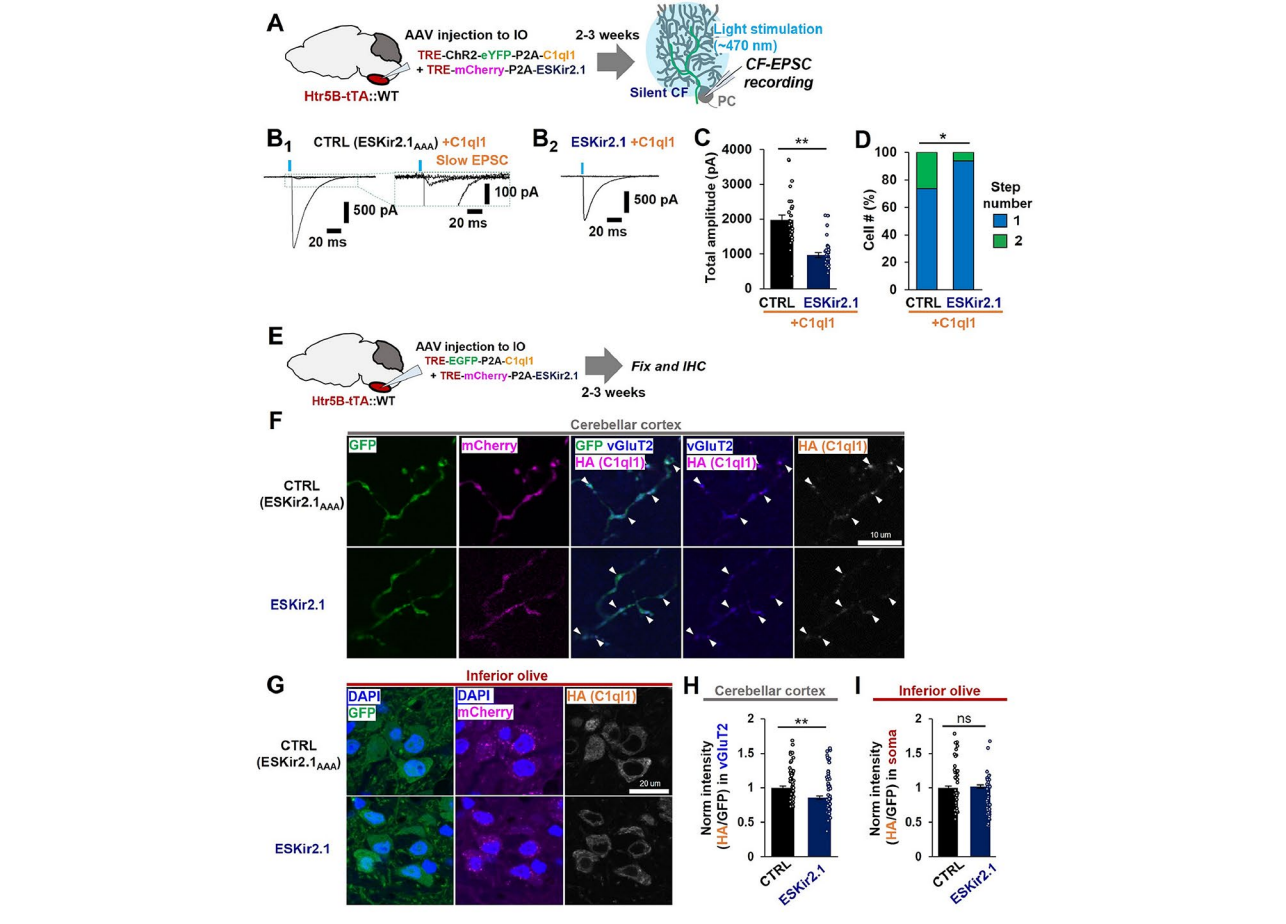
CF activity is required for C1ql1 to induce CF innervation on adult PCs **A** Experimental scheme for silencing of CFs and recording of CF-EPSC. **B** Representative light-evoked CF-EPSC traces (blue lines). Multiple EPSCs with a slower rise time were recorded in slices overexpressing C1ql1 in CFs (**B**_**1**_). The boxed region is enlarged on the right to show the slow CF-EPSC. Single EPSC was evoked in an all-or-none manner in slices overexpressing C1ql1 and ESKir2.1 in CFs (**B**_**2**_). **C** Total CF-EPSC amplitude. p = 2.665 × 10^− 7^, two-sided Welch’s t-test. n = 27 cells from 5 mice (CTRL), n = 31 cells from 5 mice (ESKir2.1). **D** The percentage of the number of CFs innervating single PCs. The number of EPSCs evoked by distinct CF activation thresholds (number of steps) is shown. p = 0.0221, Mann–Whitney U test. n = 34 cells from 5 mice (CTRL), n = 34 cells from 5 mice (ESKir2.1). **E** Experimental scheme to study the effect of CF neural activity on C1ql1 immunoreactivity. **F** Immunohistochemical analysis of HA-C1ql1 at CF synapses. Expression of ESKir2.1 (GFP, bottom panels) in IOs reduced C1ql1 (HA) immunoreactivity at CF synapses (vGluT2, arrowheads) compared to ESKir2.1_AAA_ (GFP, top panels). Scale bar, 10 μm. **G** Immunohistochemical analysis of HA-C1ql1 in IONs. Scale bar, 20 μm. **H** Quantification of HA-C1ql1 levels at CF synapses. HA-C1ql1 immunoreactivity was normalized by the GFP fluorescence in vGluT2-positive CF synapses. p = 2.353 × 10^− 4^, two-tailed Welch’s t-test. n = 103 areas from 3 mice (CTRL), n = 169 areas from 3 mice (ESKir2.1). **I** Quantification of HA-C1ql1 levels in IONs. HA-C1ql1 immunoreactivity was normalized by the GFP fluorescence in the soma of IONs. p = 0.5970, two-tailed Welch’s t-test. n = 149 cells from 3 mice (CTRL), n = 161 cells from 2 mice (ESKir2.1). Bars represent mean ± SEM. **p < 0.01; *p < 0.05; ns, not significant

Electrophysiological data were analyzed offline using Clampfit 10 (Molecular Devices). Immunohistochemical and Ca^2+^ imaging data were analyzed by using Fiji (Image J) software. All bar graphs indicate mean ± standard error of the mean. Statistical analyses were performed using Mirosoft Excel (Microsoft) and BellCurve for Excel (Social Survey Research Information Co., Ltd.). To compare the number of CFs, which has discrete variables, we used Mann-Whitney U test for two groups (Figs. 2D and H, 3D and 7D) and Kruskal-Wallis test followed by Steel test for multiple groups (Figs. 4D, 5D and 6D). For other continuous variables, we used Welch’s t-test for comparison of two groups (Figs. 1C, 2C, E, G and I, 3E and F and 7C) and two-way ANOVA followed by Dunnett test (Fig. 5C) or Tukey test (for two parameters; Fig. 6C). Since the number of mice was small (n = 4), we also used Mann-Whitney U test for Fig. 1C and confirmed the same statistical significance (*p = 0.0433) as Welch’s t-test. The sample size, p-value, and the statistical test used in each figure are also provided in figure legends.

## Results

### Selective gene expression in IONs and CFs using AAV and knock-in mice

To test the role of C1ql1-Bai3 signaling in structural changes in mature CF PC synapses, we first examined the effect of increased expression of C1ql1 on CF synapses. To avoid possible indirect effects of misexpression of C1ql1 in mossy fibers [24], we used Htr5b-tTA knock-in mice in which IONs, the origin of CFs, specifically express tTA [25]. We delivered adeno-associated virus (AAV) encoding the tetracycline response element (TRE) followed by a channel rhodopsin-2 yellow fluorescent fusion protein (ChR2-YFP) and human influenza hemagglutinin (HA)-tagged C1ql1. ChR2 was introduced to directly assess the function of CFs in the later experiments (Fig. 1A). Three weeks after injection into 3–4-week-old mice, HA-C1ql1 and YFP were detected in IONs (Fig. 1B). C1ql1 immunostaining indicates that C1ql1 expression in the soma of IONs was doubled by AAV-based expression (Fig. 1C). In the cerebellar cortex, high levels of YFP signal were detected in the molecular layer, with no evidence of misexpression in mossy fibers with rosette-like structures in the granular layer (Fig. 1D). Immunohistochemical staining for calbindin, a PC marker, and vesicular glutamate transporter 2 (vGluT2), a presynaptic marker for CFs, showed that HA-C1ql1 was localized to CF terminals along PC dendrites (Fig. 1E). These results indicate that AAV-based delivery of C1ql1 to Htr5b-tTA knock-in mice specifically and moderately increased the amount of C1ql1 in CF terminals.

### Increased C1ql1 levels in CFs induce re-innervation of mature PCs by multiple CFs

Next, we examined the effect of increased C1ql1 expression on the function of CF-PC synapses using whole-cell patch-clamp recordings from PCs in acute slices. AAV-TRE-ChR2-YFP (control) or AAV-TRE-ChR2-YFP-P2A-C1ql1 was injected into the ION of Htr5b-tTA knock-in mice at 3–4 weeks of age (Fig. 2A). Application of two light stimuli with an interval of 100 ms evoked paired-pulse depression of excitatory postsynaptic currents (EPSCs), a result consistent with a high release probability of CF terminals. Furthermore, increasing light intensity elicited EPSCs in an all-or-none manner in control slices (Fig. 2B_1_), indicating that PCs are innervated by a single CF input with a single excitation threshold. In contrast, overexpression of C1ql1 in CFs not only increased the amplitude of EPSCs, but also led to the appearance of CFs with two to three activation thresholds (Fig. 2B_2_, C, D). In addition, EPSCs evoked by different activation thresholds had a slower rise time than EPSCs evoked by most CFs (Fig. 2B_2_, E). These results suggest that increased expression of C1ql1 in CFs not only enhanced the functions of existing CF-PC synapses, but also induced new CF synapses with distinct properties.

PCs establish a mature innervation pattern with a single CF by postnatal day 20 in rodents [7]. To rule out the effect of C1ql1 overexpression on PC development, we examined PC dendritic arborization, which could affect CF synapse formation. Immunohistochemical staining of PCs with calbindin revealed no gross differences in the dendritic arborization between PCs innervated by control and C1ql1-overexpressing CFs (Supplementary Fig. 1A). In addition, C1ql1 overexpression in CFs did not affect the membrane capacitance of PCs, an electrophysiological estimate of the total surface area (Supplementary Fig. 1B). Furthermore, AAV-based overexpression of C1ql1 in 6-week-old mice increased the percentage of PCs innervated by multiple CFs in the same manner as in 3-week-old mice (Supplementary Fig. 1C, D). These results indicate that the effect of C1ql1 overexpression was not confounded by the developmental stage of the PCs.

C1ql1 regulates CF-PC synapse formation by binding to the CUB domain of Bai3 during development [18, 19]. To determine whether the effect of C1ql1 requires Bai3, we overexpressed C1ql1 in Bai3 knockout mice at 3–4 weeks of age. CF-evoked EPSC amplitudes were much smaller in Bai3 knockout than in wild-type mice (Fig. 2C vs. 2G), a result consistent with previous reports [18, 19]. In contrast to conditional knockout mice in which the Bai3 gene was postnatally deleted [18], PCs in constitutional Bai3 knockout mice did not show a multiple innervation pattern by CFs (Fig. 2H). Importantly, overexpression of C1ql1 in CFs did not result in an increase in the amplitude of CF-evoked EPSCs in Bai3 knockout PCs (Fig. 2G). In addition, overexpression of C1ql1 did not affect the percentage of PCs innervated by CFs with multiple excitation thresholds (Fig. 2F2, H). Similarly, we did not detect CF-evoked EPSCs with a slower rise time (Fig. 2F2, I). Taken together, these results suggest that C1ql1 overexpression, likely via binding to Bai3, could induce the formation of new CF synapses and increase the proportion of PCs innervated by multiple CFs in mature PCs.

### C1ql1-Bai3 signaling induces synapse formation by transverse CF branches

How can a PC in which excess CFs have already been pruned except for a dominant single CF be innervated again by other CFs? A previous in vivo time-lapse imaging study showed that while ascending branches of CFs formed stable synapses with proximal dendrites of PCs, the thin transverse branches were highly dynamic and did not make synapses in adult wild-type mice [26]. Thus, to clarify the contribution by transverse CF branches, we traced GFP-positive CFs in coronal cerebellar sections from wild-type mice to which AAV-TRE-GFP (control) or AAV-TRE-GFP-P2A-C1ql1 was injected at 3–4 weeks of age (Fig. 3A, B_1_). Co-immunostaining of GFP and vGluT2 revealed that the transverse CF branches were observed at various locations along the PC dendrites, but they mostly lacked vGluT2 in control sections (Fig. 3B_2_, C, D), indicating their inability to form functional synapses as reported previously [20, 26, 27]. Interestingly, when C1ql1 was overexpressed in CFs, transverse branches elongated and often became positive for vGluT2 (Fig. 3B_2_, C, D). The elongation of the transverse branch occurred mostly in distal dendrites (80–160 μm from the soma) (Fig. 3 C, E). Transverse branches that were positive for vGluT2 were longer than those negative for vGluT2 (Fig. 3F). Since EPSCs at synapses farther electrotonic distance from the recording site show a slower rise time, these results suggest that an increased proportion of PCs innervated by multiple CFs is at least partly caused by the transverse CF branches forming synapses on distal dendrites.

To determine whether the effect of C1ql1 on CF transverse branches required Bai3, we next traced CFs in coronal cerebellar sections from Bai3 knockout mice (Supplementary Fig. 2A) to which AAV-TRE-GFP (control) or AAV-TRE-GFP-P2A-C1ql1 was injected. In Bai3 knockout mice, the overexpression of C1ql1 in CFs did not elongate the transverse CF branch or increase the percentage of vGluT2-positive terminals (Supplementary Fig. 2B-F). These results indicate that overexpression of C1ql1, likely via interaction with Bai3, could induce the growth and synapse formation by of transverse CF branches, resulting in an increased number of PCs re-innervated by multiple CFs after CF-PC synapses mature.

### Bai3 overexpression in PCs induces re-innervation by surplus CFs through C1ql1 binding

While new CF-PC synapses were induced by overexpression of C1ql1 in CFs, it was unclear whether PCs overexpressing Bai3 could form new synapses with CFs with normal levels of C1ql1. To address this question, we used lentivirus with the murine stem cell virus (MSCV) promoter [28] to preferentially express EGFP and Bai3 in PCs of mice at 3–4 weeks of age (Fig. 4A). Bai3 expression levels were estimated to be increased by approximately 1.8-fold (Supplementary Fig. 3A, B). We recorded CF-evoked EPSCs from whole-cell patch-clamped PCs by placing the stimulating electrode in the granular layer near the PC soma. CF-EPSCs, which were confirmed by the paired-pulse depression, were elicited in an all-or-none manner in PCs expressing EGFP only, confirming that approximately 90% of wild-type PCs are innervated by a single CF input (Fig. 4 C, D). In contrast, EPSCs were evoked by two or three thresholds of stimulation in PCs overexpressing wild-type Bai3 (Fig. 4 C, D), suggesting that Bai3 overexpression in PCs induces re-innervation by surplus CFs.

To rule out an effect of Bai3 overexpression on PC development, we examined the membrane capacitance of PCs. As in the case of C1ql1 overexpression in CFs, overexpression of Bai3 in PCs did not affect the membrane capacitance of PCs (Supplementary Fig. 4A), suggesting no gross differences in the total surface area of PCs. Furthermore, lentivirus-based overexpression of Bai3 in 6-week-old mice increased the percentage of PCs innervated by multiple CFs in the same manner as injection into 3-week-old mice (Supplementary Fig. 4B, C). These results indicate that the effect of Bai3 overexpression was not confounded by the developmental stage of the PCs.

Bai3 belongs to the adhesion G protein-coupled receptor family, which mediates intracellular signaling through distinct functional domains [29–32]. To gain insight into the signaling mechanism mediated by Bai3, we expressed Bai3 with mutations in these functional domains (Fig. 4B). Expression of Bai3-AAA, disabling the ELMO binding motif [29, 30], Bai3-ΔCT7, which lacked the PDZ binding motif [32] and Bai3-S832A, which disrupted the proteolysis sequence in the GPCR auto-proteolysis-inducing (GAIN) domain [31], had similar effects as wild-type Bai3 in inducing re-innervation of PCs by surplus CFs (Fig. 4 C, D). In contrast, the expression of Bai3-ΔCUB, which lacked the binding site for C1ql1 [18], did not affect the pattern of CF innervation in mature PCs (Fig. 4 C, D). These results indicate that overexpression of Bai3 in mature PCs induced innervation by additional CFs by binding to C1ql1, but independently of ELMO, PDZ proteins or proteolysis at the GAIN domain.

### Bai3 overexpression in PCs induces re-innervation by CFs at distal dendrites

The largest EPSCs observed in PCs overexpressing Bai3, which we termed “main CF-EPSC”, had similar kinetics to EPSCs seen in control PCs, but the smaller EPSCs (surplus CF-EPSCs) elicited by distinct stimulus thresholds had slower rise times (Fig. 4E). Since overexpression of C1ql1 in CFs also caused the appearance of small CF-evoked EPSCs with slower kinetics (Fig. 2E) and synapse formation at distal dendrites by transverse CF branches (Fig. 3C), we hypothesized that Bai3 overexpression in PCs similarly induced new CF synapse formation on distal dendrites.

To test this hypothesis morphologically, we expressed EGFP and Bai3 in PCs by lentivirus and sparsely labeled IONs by injecting AAV-TRE-tdTomato into Htr5b-tTA knock-in mice (Supplemental Fig. 3C). We found a few PCs that expressed Bai3 and were selectively innervated by transverse CF branches expressing tdTomato without labeled main CF inputs (Supplemental Fig. 3D). However, since the identification of surplus CF branches relies on the coincidental sparse labeling of PCs and CFs, it was difficult to quantify the effect of Bai3 on the formation of surplus CF synapses by the immunohistochemical method.

To clarify the location of surplus CF synapses that gave rise to EPSCs with slow kinetics, we next used the electrophysiological mapping method. We systematically moved the stimulating electrode every 10 μm in the XY direction in the granular layer (Supplementary Fig. 5A). We found that at some locations, main and surplus EPSCs could be evoked by varying the stimulus intensity, while at other locations, only main or surplus EPSCs were selectively evoked. Overall, the location of the stimulating electrode that evoked surplus EPSCs was farther from the PC soma than that elicited main EPSCs (Supplementary Fig. 5B). These results suggest that in PCs overexpressing Bai3, surplus EPSCs are evoked by CFs that travel farther from the cell body than the main CF.

To directly visualize where surplus CFs formed functional synapses with PCs overexpressing Bai3, we loaded PCs with a Ca^2+^ indicator (Oregon green BAPTA-1) through a patch electrode. We first identified the sites where only main or surplus CF-EPSCs were selectively evoked (Supplementary Fig. 5C) and then performed Ca^2+^ imaging under the current-clamp mode (Fig. 4F-I). Stimulation of sites where main EPSPs were selectively elicited caused greater increases in Ca^2+^ concentrations from a larger dendritic area than stimulation of sites where surplus EPSPs were selectively elicited (Fig. 4G, H). Furthermore, Ca^2+^ elevations associated with surplus EPSPs were observed in dendrites more distal to the PC soma than those associated with the main EPSPs (Fig. 4G, I). These results further support the hypothesis that overexpression of Bai3 in PCs causes the formation of new CF synapses on dendrites more distal to the main CFs, resulting in multiple CF innervation of mature PCs.

During development, the inhibitory inputs from molecular layer interneurons and CFs compete for synapses on PC somata [33]. In GluD2 knockout mice, PFs and CFs compete for synapses on distal dendrites of PCs [20]. Therefore, to explore the possibility that Bai3 may affect other types of PC synapses, we recorded PF-evoked EPSCs, which were confirmed by paired-pulse facilitation, in PCs overexpressing Bai3 (Supplementary Fig. 6A). The amplitudes of PF-EPSCs in response to increasing stimulus intensities were similar between PCs expressing EGFP only (control) and EGFP plus Bai3 (Supplementary Fig. 6B, C). Miniature inhibitory postsynaptic currents (mIPSCs) recorded from PCs overexpressing Bai3 Supplementary Fig. 6D) and control showed similar amplitudes and frequencies (Supplementary Fig. 6E, F). Although local competition may be missed because PF and inhibitory synapses outnumber CF synapses, these results indicate that overexpression of Bai3 in mature PCs preferentially induces CF synapses without significantly altering the number of other synapses.

### Endogenous Bai3 and C1ql1 are involved in the re-innervation of CFs in mature PCs

Can CFs form new synapses in mature cerebellar circuits that do not overexpress C1ql1 or Bai3? Loss of PF-PC synapses in conditional GluD2 knockout mice has been reported to trigger re-innervation of PCs through CF transverse branches without exogenous manipulation of C1ql1-Bai3 signaling [20]. Therefore, we investigated the role of endogenous Bai3 in conditional GluD2 knockout mice. Using lentivirus with an MSCV promoter, we sparsely expressed a Cre recombinase and EGFP in PCs of 3–4-week-old wild-type and conditional GluD2 (*Grid*2^*f/f*^) and/or Bai3 (*Bai3*^*f/f*^) knockout mice (Fig. 5A, Supplementary Fig. 7A). Whole-cell patch-clamp recordings from acute cerebellar slices prepared from conditional GluD2 knockout mice two months after Cre introduction revealed that PCs were innervated by multiple CFs with distinct thresholds (Fig. 5B, D), as previously reported [20]. In contrast, CF evoked smaller EPSCs in an all-or-none manner in conditional Bai3 knockout mice (Fig. 5B-D), indicating that the pattern of innervation of PCs by a single CF is unaffected by knocking out Bai3 in adult mice as reported previously [12]. Interestingly, in contrast to GluD2 knockout mice, when both GluD2 and Bai3 were knocked out, CF-EPSCs became smaller (Fig. 5B, C), but many PCs remained innervated by a single CF (Fig. 5D). Furthermore, C1ql1 immunopositive puncta were significantly upregulated in the upper molecular layer of GluD2 knockout mice at 2–3 months of age (Supplemental Fig. 7B, C, D), suggesting the involvement of C1ql1 in CF synapse formation in GluD2 knockout mice. These results suggest that endogenous Bai3, probably together with endogenous C1ql1, is required for re-innervation of mature PCs by CFs in GluD2 knockout mice.

### Bai3-induced re-innervation of PCs by CFs requires PC activity

Since structural synaptic plasticity occurs in an activity-dependent manner throughout life in the mammalian brain [34, 35], we next investigated whether increased C1ql1-Bai3 levels could bypass neuronal activity to form new CF synapses in mature PCs. Using an AAV-based Cre-DIO (double-floxed inverse open reading frame) system, we expressed EGFP and ESKir2.1, a non-rectifying variant of Kir2.1 potassium channel [36], to specifically suppress levels of intrinsic PC activity (Supplementary Fig. 8A). As a control, we used ESKir2.1_AAA_, a mutant channel lacking channel activity [36]. Loose-patch recordings in acute cerebellar slices prepared from mice 2–3 weeks after the AAV injection at 3–4 weeks of age confirmed the absence of spontaneous action potentials in PCs expressing ESKir2.1, but not ESKir2.1_AAA_ (Supplementary Fig. 8B, top traces). Whole-cell voltage-clamp recordings revealed that the amplitude of CF-evoked EPCSs was reduced in PCs expressing ESKir2.1, but the number of stimulus thresholds (reflecting the number of CF inputs) was similar to control PCs (Supplementary Fig. 8B-D). Similarly, the application of tetrodotoxin or NBQX is reported to reduce the amplitude of CF-EPSCs and CF synapses in adult PCs [11–13]. While the site of action was unclear in these pharmacological studies, our findings indicate that the intrinsic activity of PCs is required to maintain CF synapses in mature PCs.

Next, we examined whether Bai3 overexpression could induce CF re-innervation in PCs expressing ESKir2.1 by coinfecting L7-Cre mice with AAV-Syn-DIO-ESKir2.1-T2A-EGFP and Lenti-MSCV-mCherry-P2A-Bai3 (Fig. 6A). In PCs overexpressing Bai3 and ESKir2.1_AAA_, we detected CF-EPSCs with multiple thresholds (Fig. 6B, left traces; Fig. 6D), as observed in the absence of ESKir2.1_AAA_ (Fig. 4 C, D). In contrast, CF stimulation evoked smaller EPSCs with a single threshold in most PCs coexpressing ESKir2.1 and Bai3 (Fig. 6B, middle traces; Fig. 6D). These results indicate that intrinsic PC activity is required for Bai3 to re-innervate mature PCs.

In these experiments, expression of ESKir2.1 (as detected by EGFP) was widespread in many PCs at the injection site, whereas Bai3 (as detected by mCherry) was detected in only a few PCs (Supplementary Fig. 8E). However, we occasionally found non-silenced PCs expressing only Bai3, surrounded by silent PCs expressing only ESKir2.1 (Fig. 6A, right; Supplementary Fig. 8E, bottom). The amplitude of CF-EPSCs in such non-silenced PCs was similar to that in control PCs expressing ESKir2.1_AAA_ and Bai3 (Fig. 6C). Unexpectedly, however, CF-EPSCs were evoked by a single threshold in these non-silenced PCs (Fig. 6B, right traces; Fig. 6D). These results suggest that multiple innervation by CFs requires neuronal activity not only in the Bai3-expressing PCs but also in the surrounding PCs.

### CF activity is required for C1ql1 to induce the innervation of adult PCs by CFs

Finally, we investigated whether increased C1ql1 levels in CFs could induce new CF synapses in the absence of CF activities. To suppress CF activities in vivo, we injected a mixture of AAV-TRE-ChR2-YFP and AAV-TRE-mCherry-P2A-ESKir2.1 into the ION of Htr5B-tTA knock-in mice at 3–4 weeks of age (Supplementary Fig. 9A). With acute slice preparations where input fibers are not preserved, it is difficult to determine how effectively ESKir2.1 could silence the electrical activity of IONs *in vivo.* Instead, we injected harmaline intraperitoneally, which transiently increases the synchronous firing of IONs [26], and performed immunohistochemical staining for c-Fos, a marker of neuronal activity, 10 min after the injection (Supplementary Fig. 10A). We found that the number of c-Fos-positive IONs was significantly reduced by the expression of ESKir2.1 compared to ESKir2.1_AAA_ (Supplementary Fig. 10B, C). In addition, the amplitude of CF-EPSCs was decreased by the expression of ESKir2.1 in IONs (Supplementary Fig. 9B, C) while the innervation pattern of CF was unchanged (Supplementary Fig. 9B, D), indicating that the intrinsic activity of IONs, which is required to maintain CF synapses in adult PCs, was suppressed by ESKir2.1.

To examine the effect of CF activities on C1ql1-induced CF synapse formation, we next injected a mixture of AAV-TRE-ChR2-YFP-P2A-C1ql1 and AAV-TRE-mCherry-P2A-ESKir2.1 into the ION of Htr5B-tTA knock-in mice (Fig. 7A). Whole-cell patch-clamp recordings revealed that in mice expressing ChR2, C1ql1 and ESKir2.1_AAA_ were expressed in the IONs, additional EPSCs with a slow time course were elicited in response to an increasing light stimulus (Fig. 7B_1_), as observed in the absence of ESKir2.1_AAA_ (Fig. 2B_2_). In contrast, when C1ql1 was overexpressed with ESKir2.1 in the IONs, the amplitude of CF-EPSCs was significantly reduced (Fig. 7B_2_, C). Furthermore, the percentage of PCs that were innervated by the surplus CFs was reduced by co-expression of C1ql1 and ESKir2.1 (Fig. 7D). These results indicate that CF activity is required for C1ql1 to induce additional CF innervation onto adult PCs.

To gain insight into why CF activity enhances synaptogenesis through C1ql1, we performed immunohistochemical staining of HA-C1ql1 in mice expressing HA-C1ql1 and either ESKir2.1 or ESKir2.1_AAA_ in the IONs (Fig. 7E). In the cerebellar cortex, HA-Clql1 immunoreactivity on vGluT2-positive CF terminals was significantly reduced in CFs expressing ESKir2.1 as compared to those expressing ESKir2.1_AAA_ (Fig. 7F, H). In contrast, no difference was observed in HA-C1ql1 immunoreactivity in the cell bodies of IONs expressing ESKir2.1 versus ESKir2.1_AAA_ (Fig. 7G, I). While the neuronal activity of IONs could affect CF synaptogenesis through various pathways (discussed in detail below), our findings suggest that one possibility is its involvement in the secretion from CF terminals since C1ql1 immunoreactivity in the adult cerebellar cortex is detected in the synaptic cleft [18].

## Discussion

**Fig. 8:**
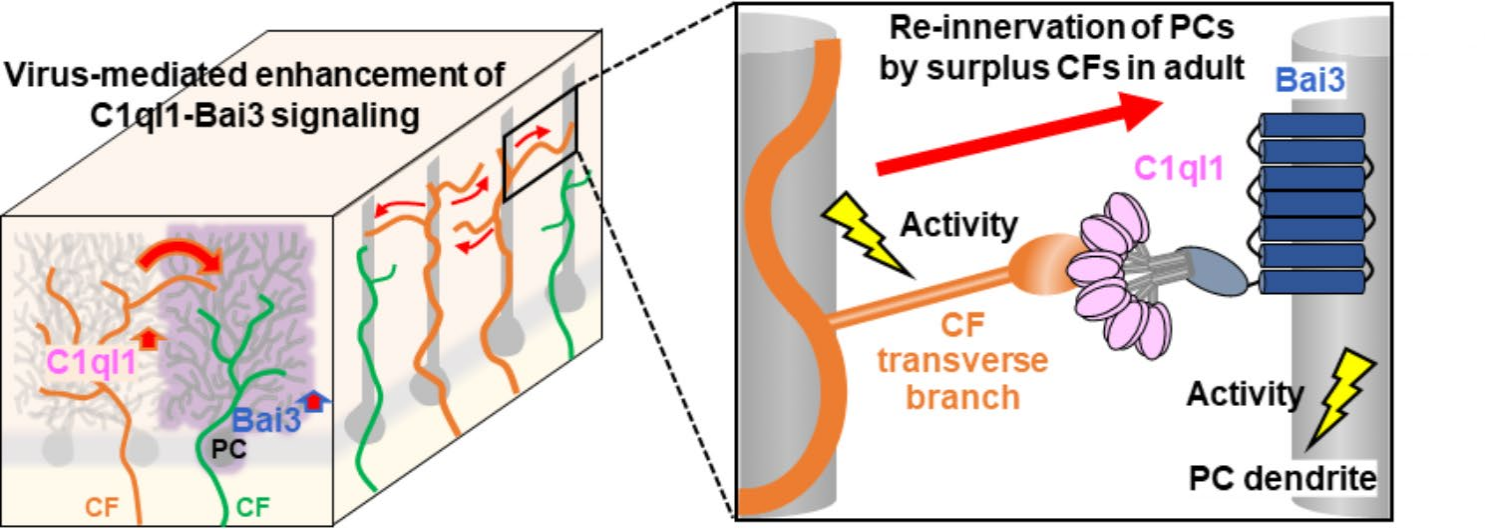
Proposed model for re-innervation of mature PCs by excess CFs through C1ql1-Bai3 signaling Increased C1ql1-Bai3 signaling induces re-innervation of mature PCs by excess CFs. These new CF inputs, derived from transverse CF branches of neighboring PCs, synapse onto the distal dendrites of PCs (left). Neuronal activity of both CFs and PCs is required for C1ql1-Bai3 signaling to induce surplus CFs (right)

In the present study, we showed that mature PCs, which achieved innervation by a single strong CFs after pruning weak CFs during development, became re-innervated by surplus CFs when the expression of C1ql1 or Bai3 was upregulated in CFs or PCs, respectively. Immunohistochemical (Fig. 3E, Supplementary Fig. 3D), electrophysiological (Figs. 2E and 4E), and Ca^2+^ imaging (Fig. 4G, I) studies indicated that transverse CF branches most likely contributed to the formation of surplus CF synapses at distal dendrites of mature PCs. The effect of C1ql1 overexpression in CFs required normal levels of Bai3 in PCs (Fig. 2H, Supplementary Fig. 2 C, D). In addition, the effect of overexpression of Bai3 in PCs required the CUB domain, a binding site of Bai3 for C1ql1 (Fig. 4D). Although C1ql1 and Bai3 have additional binding partners, such as kainate receptors [37] and RTN4 [38], respectively, these results indicate that CF-derived C1ql1 binds to Bai3 in PCs to induce the formation of new CF synapses. Interestingly, endogenous Bai3 was required for the re-innervation of mature PCs by CFs in GluD2 knockout mice (Fig. 5D). Furthermore, the effect of C1ql1-Bai3 signaling on CF innervation required neuronal activity of both PCs (Fig. 6D) and CFs (Fig. 7D). Together, we propose a model in which C1ql1-Bai3 signaling mediates CF structural plasticity in mature PCs in a manner dependent on neuronal activity (Fig. 8).

### Activity-dependent effect of C1ql1 and Bai3 on re-innervation of mature PCs by CFs

Why do C1ql1 and Bai3 require neuronal activity in both PCs and IONs to induce CF synapses in mature PCs? P/Q-type Ca^2+^ channels and aCaMKII regulate the elimination of surplus CFs during development since PCs genetically lacking these molecules remain innervated by surplus CFs in adulthood [41–43]. However, the selective strengthening of the winner CFs is also impaired in PCs lacking Ca^2+^ influx through P/Q Ca^2+^ channels [44], indicating that Ca^2+^ influx is not only required to eliminate weak CFs, but also to selectively strengthen strong CFs during development. Thus, activity-induced Ca^2+^ influx in PCs may play a role in maintaining newly formed CF synapses in mature PCs.

Since C1ql1 immunoreactivity at CF-PC synapses was reduced when the activity of IONs was suppressed (Fig. 7H), C1ql1 may be released from CFs in an activity-dependent manner. Similarly, the C1q family protein Cbln1 is released from PFs in an activity-dependent manner [21]. It has been reported that the motility of transverse CF branches lacking vGluT2-positive presynaptic sites was reduced 3 h after the application of harmaline [26]. Thus, the activity-induced Ca^2+^ increase in CF transverse branches may slow down their motility to facilitate the accumulation of vesicles containing synapse organizers, such as C1ql1, to facilitate synapse formation with mature PCs. In addition, changes in the extracellular matrix (ECM) associated with CF activity may allow C1ql1 to be stabilized at synapses. Indeed, activity-dependent release of cathepsin B, a lysosomal enzyme co-released with Cbln1, allows presynaptic morphological changes associated with PF-PC synapse formation [21]. Back-propagating action potentials also trigger the exocytosis of cathepsin B from dendrites and subsequent activation of matrix metalloproteinase 9, an enzyme involved in ECM remodeling, leading to activity-dependent spine growth in hippocampal neurons [45]. Since innervation by surplus CFs required neural activity not only in the Bai3-expressing PCs but also in the surrounding PCs, activity-dependent modification of the ECM may provide a permissive environment for new synapse formation in mature PCs.

### C1ql1-Bai3 exerts synaptogenic function through uncharacterized domains

Bai3 is reported to inhibit dendritogenesis of PCs during development by regulating the activity of the small GTPase Rac1 through the interaction with ELMO1 and DOCK180 [29]. Similarly, Bai3 has been shown to mediate the fusion of myoblasts by binding to ELMO1/DOCK1 during development [30]. Bai1, a close relative of Bai3, is reported to promote synaptogenesis through the PDZ binding motif by associating with Nlgn1 [46], recruiting the Rac1-GEF complex Par3/Tiam1 [47] and stabilizing PSD-95 [48]. An engineered truncation in Bai1 and Bai3, mimicking autocleavage at the GAIN domain, led to activation of Ga_12/13_ [32] and G_ai1_ [30], respectively. However, in the present study, mutations disrupting the ELMO1 binding motif, the PDZ binding motif and the GAIN domain did not affect the ability of Bai3 to induce CF synapses in adult mice (Fig. 4D). Since mutant Bai3 was expressed in wild-type mice, the mutant Bai3 may have associated with endogenous Bai3 to compensate for the function of the mutated site. However, the inability of Bai3-ΔCUB to induce surplus CF innervation indicates that at least the CUB domain defect cannot be compensated for by endogenous Bai3. Thus, considering that Bai1 can mediate at least five downstream signaling pathways by differentially coupling to ELMO, MDM2, Par3/Tiam1, Ga_12/13_ and Bcr, depending on the cellular context [49], we postulate that C1ql1 binding to Bai3 likely exerts its synaptogenic function through distinct domains that interact with uncharacterized signaling pathways.

### Functional implication of added CF synapses by transverse branches

It is difficult to chronically increase the activities of IONs in vivo to investigate whether the expression of endogenous C1ql1 or Bai3 changes and induces re-innervation by CFs. For example, although harmaline administration rapidly induces tremor-like movements in rodents associated with increased activity of IONs, the effect is transient and lasts only a few days [50]. However, knockout of GluD2 caused re-innervation of mature PCs by multiple CFs without overexpression of C1ql1 or Bai3, but only in the presence of endogenous Bai3 in PCs (Fig. 5D). Importantly, C1ql1 immunopositive puncta were upregulated in adult GluD2 knockout mice (Supplemental Fig. 7B, C, D). These findings suggest that endogenous C1ql1-Bai3 signaling is involved in the re-innervation of PCs by CFs under certain pathological conditions.

The main branches of CFs are distributed parasagittally in microzones [51], which likely contain ~ 100 PCs in mice [52]. In contrast, CFs mediolaterally extend transverse branches for 5–300 μm without forming synapses in adult mice [26, 53]. Many PCs belonging to the same microzone show synchronous activity due to electrical coupling between IONs. A computer simulation study indicates that the higher level of electrical coupling of IONs accelerates the crude learning at the initial stage by facilitating the synchronized firing of PCs, while the reduced electrical coupling at the later stage of learning allows more sophisticated and complicated learning [54]. Alternatively, synchronized firing by enhanced electrical coupling of IONs may represent a state change underlying skilled movements [55]. Interestingly, PCs in GluD2 knockout mice are reported to show enhanced synchronous firing in the mediolateral direction, which was not mediated by electrical coupling of IONs, but by surplus transverse CF branches making synapses onto distal PC dendrites in vivo [56]. Similarly, after partial lesion of IONs, surviving CFs are reported to sprout new collaterals in the mediolateral direction and innervate PCs [57]. A live imaging study showed that these newly sprouted transverse CF branches preferentially synapse on PCs near the target of the original PCs [58]. Thus, although the EPSCs and Ca^2+^ transients elicited by newly formed transverse CF synapses are generally small (e.g., Fig. 2B_2_, 4 F, G), they can cause synchronous firing of adjacent PCs in the mediolateral direction, thereby affecting cerebellar learning or recovery after injury.

Cerebellar microzones show differential immunoreactivity to zebrin II, largely corresponding to a distinct ensemble of PCs with similar functional properties [59]. However, transverse CF branches induced by partial ION lesions [58] or in GluD2 knockout mice [56] can partially cross the zebrin II boundary, leading to the connection of some PCs belonging to different microzones. Thus, the physiological role of newly formed synapses by CF transversal branches could be complicated depending on the extent to which CF synapses are formed across the zebrin II boundary. Since C1ql3, a closely related family member of C1ql1, and Bai3 are expressed in other neural circuits, such as the basolateral amygdala-medial prefrontal cortex [60] and the anterior olfactory neuron-olfactory bulb [61], further studies are warranted to clarify whether and how C1ql-Bai3 signaling mediates activity-dependent structural plasticity in these brain regions in adulthood.

## Electronic supplementary material

Below is the link to the electronic supplementary material.

Supplementary Material 1

## Data Availability

The datasets and plasmids used in this study are available from the corresponding author on reasonable request.

## Abbreviations

AAV: Adeno-associated virus
AMPA: α-amino-3-hydroxy-5-methyl-4-isoxazolepropionic acid
Bai3: Brain specific angiogenesis inhibitor 3
C1ql1: C1q-like protein 1
Cbln1: Cerebellin-1
CF: Climbing fiber
ChR2: Channel rhodopsin-2
DIO: Double-floxed inverse open reading frame
EPSC: Excitatory postsynaptic currents
GluD2: Delta-type glutamate receptor 2
HA: Human influenza hemagglutinin
ION: Inferior olive neurons
mIPSC: Miniature inhibitory postsynaptic currents
MSCV: Murine stem cell virus
NBQX: 2,3-Dioxo-6-nitro-1,2,3,4- tetrahydrobenzo[f]quinoxaline-7-sulfonamide
PC: Purkinje cell
PF: Parallel fiber
TRE: Tetracycline response element
tTA: Tetracycline transactivator
vGluT2: Vesicular glutamate transporter 2
