# Supplementary material for: C1ql1-Bai3 signaling is necessary for climbing fiber synapse formation in mature Purkinje cells in coordination with neuronal activity
Source: Mol Brain. 2023 Jul 24;16:61. doi: 10.1186/s13041-023-01048-4 (PMC10367388; doi:10.1186/s13041-023-01048-4)
Supplement: Supplementary file 1 — Supplementary Material 1 [file 13041_2023_1048_MOESM1_ESM.pdf]

Additional file 1:

Supplementary Fig.1
Supplementary Fig.2
Supplementary Fig.3
Supplementary Fig.4
Supplementary Fig.5
Supplementary Fig.6
Supplementary Fig.7
Supplementary Fig.8
Supplementary Fig.9
Supplementary Fig.10
Supplementary method

C1ql1-Bai3 signaling is necessary for climbing fiber synapse formation in mature Purkinje cells in coordination with neuronal activity

Takahiro Aimi, Keiko Matsuda and Michisuke Yuzaki*

Department of Physiology, Keio University School of Medicine, Tokyo 160-8582, Japan

*Correspondence: myuzaki@keio.jp

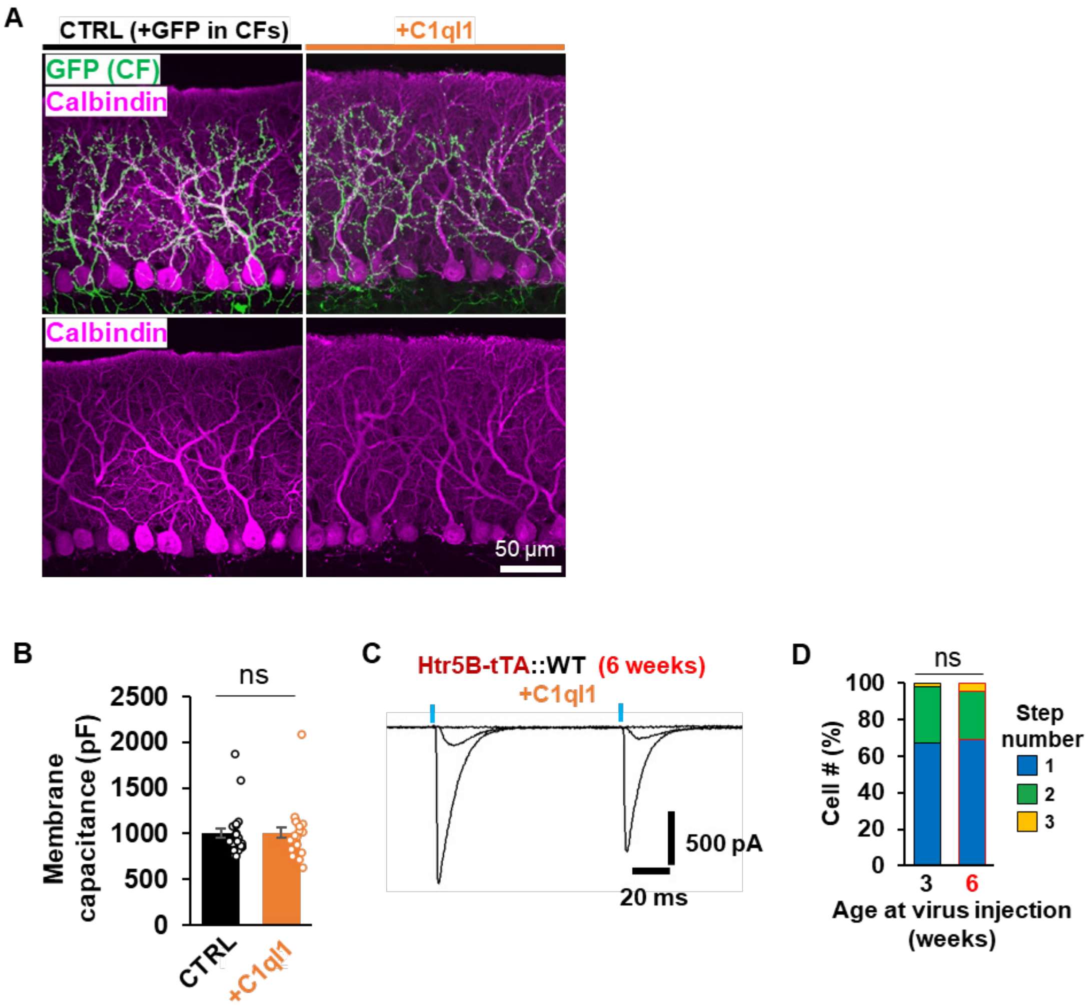

Supplementary Fig. 1, related to Fig. 2: The effect of C1ql1 overexpression on re-innervation of mature PCs by excess CFs is not mediated by PC development.

A Representative immunohistochemical images of PCs (calbindin) and CFs (GFP). GFP (CTRL) or GFP plus C1ql1 (+C1ql1) was overexpressed in the IONs. **B** Membrane capacitance of PCs in Fig.2C-E measured by the whole-cell patch-clamp recordings. p = 0.8368 by two-tailed Welch's t-test, n = 25 cells (CTRL) from 4 mice, n = 24 cells (+C1ql1) from 5 mice. Bars represent mean ± SEM. ns, not significant. **C** Representative traces of CF-EPSCs. C1ql1 overexpression was induced in 6-week-old mice. **D** Comparison of the number of steps of CF-EPSCs between the ages of AAV injection. There is no difference between 3 and 6 weeks (p = 0.9375, Mann-Whitney U-test). n = 49 cells from five 3-week mice; n = 42 cells from four 6-week mice.

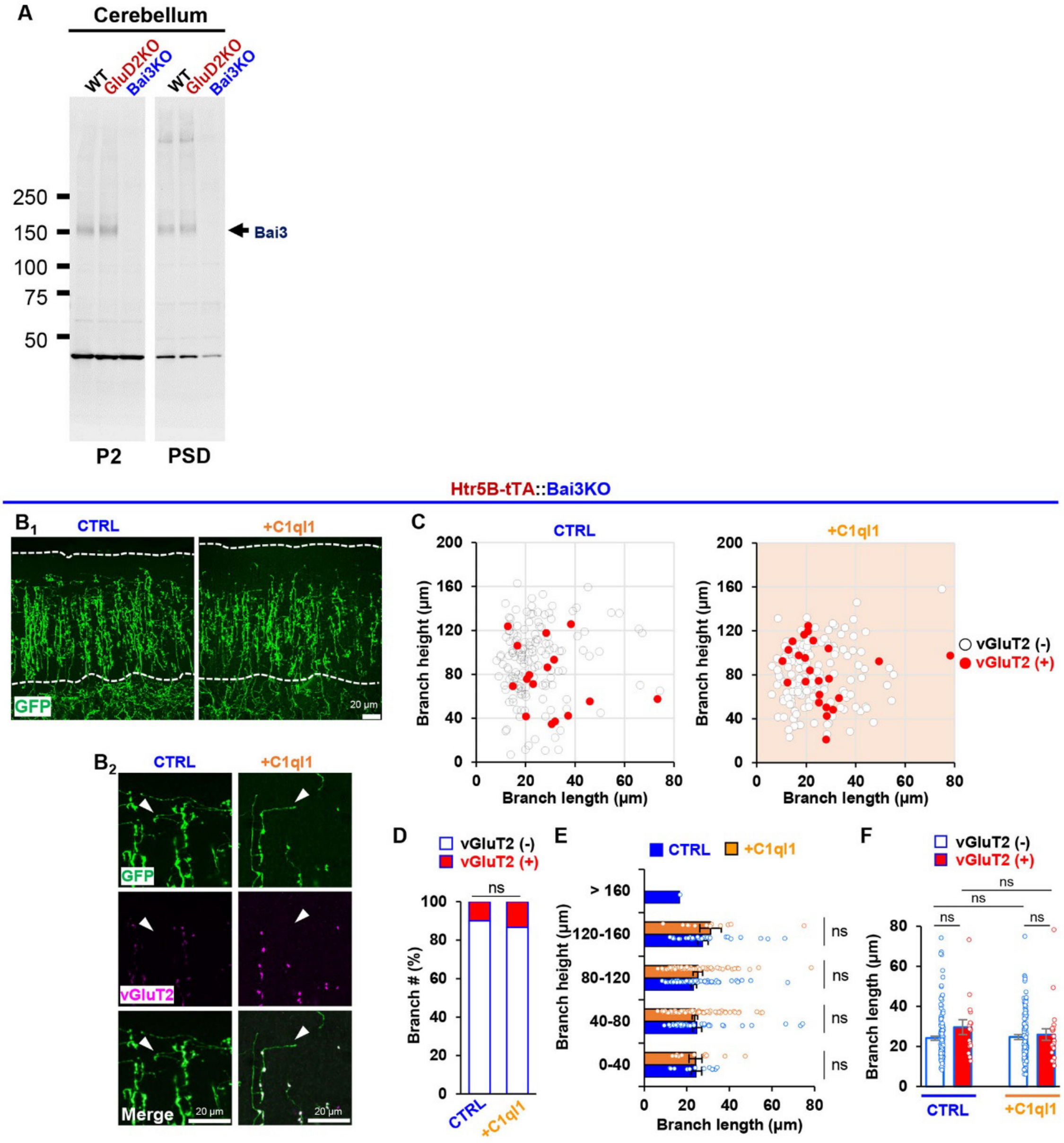

Supplementary Fig. 2, related to Fig. 3: The effect of C1ql1 overexpression in Bai3 knockout mice.

A Coronal sections showing the distribution of CF transverse branches in the Bai3 knockout (KO) cerebellum. GFP (control) or GFP plus C1ql1 (+C1ql1) was overexpressed in the CFs. Maximum intensity z-projection images are shown. Dotted lines indicate the upper and lower boundaries of the molecular layer. Scale bar, 20 µm (**A$_1$**). Enlarged views of representative CF branches (**A$_2$**). Maximum intensity z-projection images indicate vGluT2-negative branches (arrowheads). Scale bar, 20 µm. **B** Height and length of CF transverse branches in the molecular layer of Bai3 KO mice. Detailed information is provided in **C**. n = 162 branches from 4 mice (CTRL), n = 180 branches from 3 mice (+C1ql1). **C** Percentage of vGluT2-positive CF transverse branches in Bai3 KO mice expressing GFP

only (control) and GFP plus C1ql1 (+C1ql1). p = 0.3177, Mann–Whitney U test; n = 162 from 4 mice (CTRL); n = 180 from 3 mice (+C1ql1). **D** Histogram showing the mean length of CF transverse branches as a function of their height in the molecular layer of Bai3 KO mice. Orange and blue bars represent the cerebellum expressing GFP only (CTRL) and GPF plus C1ql1 (+C1ql1), respectively. Statistical analysis was omitted at >160 μm due to insufficient branching in Bai3KO. 0-40 μm: p = 0.7013, n = 12 (CTRL), n = 19 (+C1ql1); 40-80 μm: p = 0.7120, n = 49 (CTRL), n = 121 (+C1ql1); 80-120 μm: p = 0.5903, n = 68 (CTRL), n = 107 (+C1ql1); 120-160 μm: p = 0.6045, n = 31 (CTRL), n = 15 (+C1ql1). Two-tailed Welch's t-test. **E** Histogram showing the mean length of CF transverse branches by the presence or absence of vGluT2 in Bai3 KO cerebellum. There were no differences in the transverse branch length between vGluT2-negative (-) and positive (+) CFs in control mice [p = 0.1641; vGluT2(-), n = 146; vGluT2(+), n = 16] and C1ql1-expressing mice [p = 0.6972; vGluT2(-), n = 156; vGluT2(+), n = 24]. Two-tailed Welch's t-test. Bars represent mean ± SEM. ns, not significant.

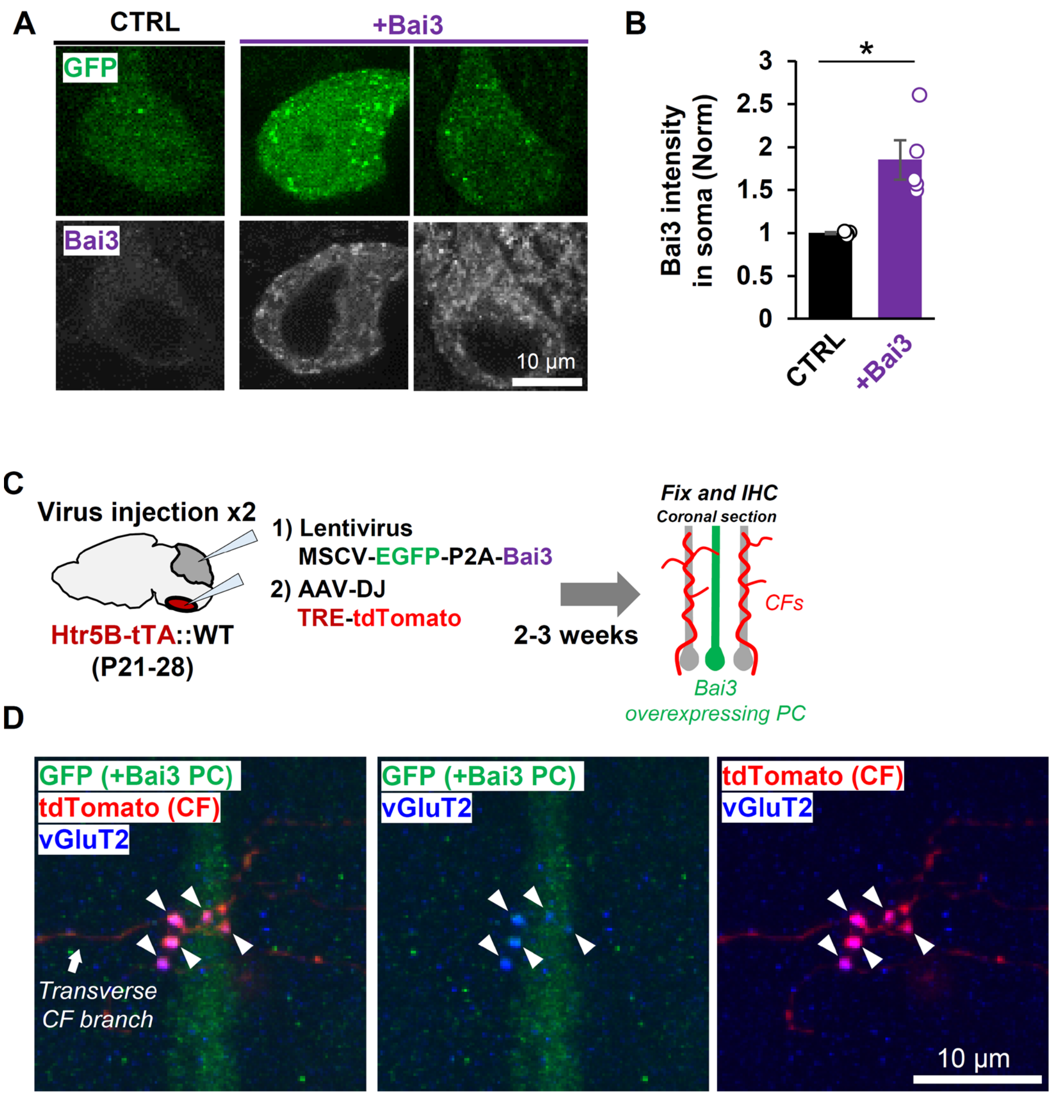

Supplementary Fig. 3, related to Fig. 4: Effect of Bai3 overexpression in PCs analyzed by immunohistochemistry.

A Representative images of Bai3 immunoreactivity in the soma of PCs in mice expressing GFP only (CTRL) or GFP and Bai3 (+Bai3). **B** Quantification of Bai3 immunoreactivity in the soma of PCs. *p = 0.0142, two-side Welch's t-test; n = 5 mice each. Bars represent mean ± SEM. **C** Experimental scheme. Bai3 overexpression in PCs was induced by lentivirus injection to the cerebellar cortex. CF labeling with another fluorescence protein (tdTomato) was induced by AAV injection into the ION. **D** An image showing vGluT2-positive and tdTomato-labeled synapses formed by transverse CF branches on the dendrites of Bai3-overexpressing PC. Scale bar, 10 µm.

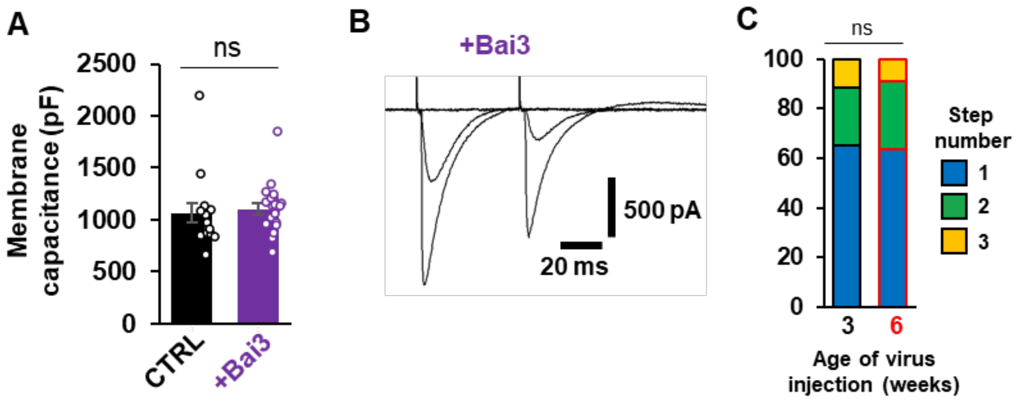

Supplementary Fig. 4, related to Fig. 4: The effect of Bai3 overexpression on re-innervation of mature PCs by excess CFs is not mediated by PC development.

A Bai3 overexpression did not affect the membrane capacitance of PCs. $p = 0.7386$, $n = 15$ cells (CTRL) from 6 mice, $n = 22$ cells (+Bai3) from 11 mice, two-tailed Welch's t-test. Bars represent mean ± SEM. **B** Representative traces of main CF-EPSC and surplus CF-EPSC recorded from PCs 2-3 weeks after the injection to 6-week-old mice. **C** Lentivirus injection into 6-week-old mice increased the percentage of PCs multiply innervated by CFs in the same manner as the injection into 3-week-old mice ($p = 0.9595$, Mann-Whitney U-test). $n = 69$ cells from eleven 3-week-old mice; $n = 33$ cells from five 6-week-old mice. ns, not significant.

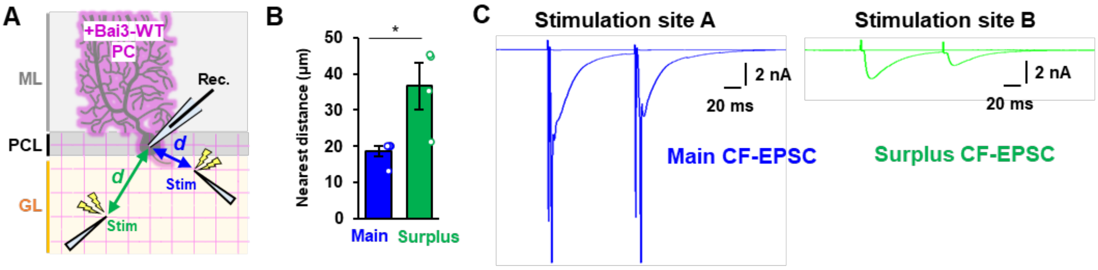

Supplementary Fig. 5, related to Fig. 4: Electrophysiological mapping of CF axons giving rise to main and surplus EPSCs in Bai3-overexpressing PCs.

A Diagram showing how the locations of stimulus electrodes to evoke surplus and main EPSCs were determined. The stimulating electrode was systematically moved every 10 mm in the XY direction in the granular layer. The distance (*d*) of the stimulating electrode from the PC soma was measured. **B** The mean distance of the stimulating electrode to evoke main and surplus EPSCs. $p = 0.0448$, two-tailed Welch's t-test, $n = 5$ traces (main), $n = 4$ traces (surplus) from 2 mice. **C** EPSCs were recorded from voltage-clamped PCs to determine the location of the stimulating electrode that selectively evoked main (site A, blue) and surplus (site B, green) CF-evoked responses. Representative EPSCs show paired-pulse depression and all-or-none responses, confirming that CFs were selectively stimulated. Then, Ca^{2+} imaging was then performed in the current clamp mode by stimulating sites A or B. Note that the stimulation at site A evoked the action potential from an unclamped region of PCs. In the current-clamp condition, both main and surplus CFs were associated with action potentials

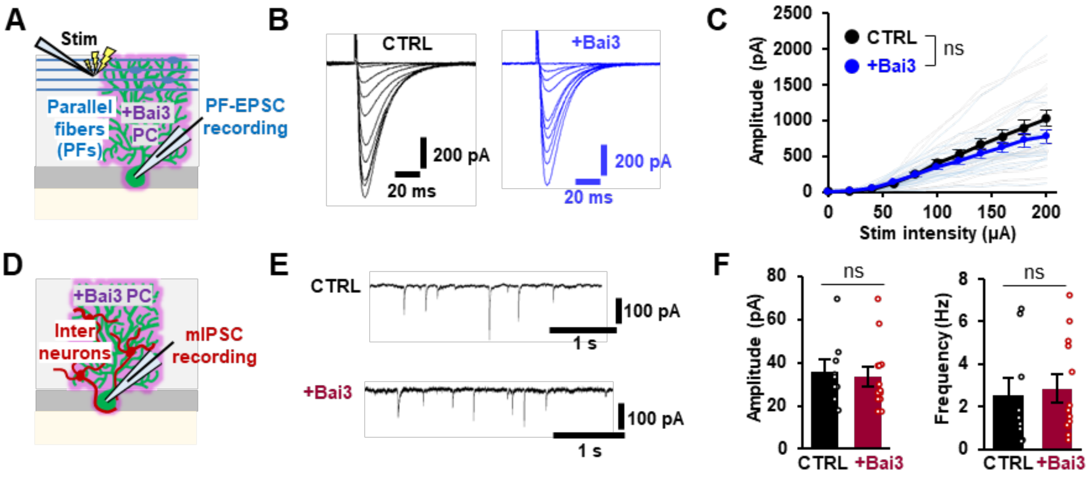

Supplementary Fig. 6, related to Fig. 4: The effect of Bai3 overexpression on PF synapses and inhibitory inputs to PCs.

A Diagram showing PF-evoked EPSC recordings from PCs overexpressing Bai3. **B** Representative PF-EPSC traces from CTRL and PCs overexpressing Bai3. PF-EPSCs were evoked by increasing stimulus intensities (0-200 mA, 20-μA increments). **C** Averaged input-output relationship of PF–EPSCs in CTRL and PCs overexpressing Bai3. p = 0.1022-0.8336, two-tailed Welch's t-test, n = 18 cells from 4 mice (CTRL), n = 19 cells from 4 mice (+Bai3). **D** Diagram showing mIPSC recording from PCs overexpressing Bai3. **E** Representative mIPSC traces from CTRL and PCs overexpressing Bai3. **F** Mean amplitude (left) and frequency (right) of mIPSCs recorded from CTRL and PCs overexpressing Bai3. p = 0.7169 (amplitude), p = 0.7553 (frequency), two-tailed Welch's t-test, n = 9 cells from 3 mice (CTRL), n = 13 cells from 4 mice (+Bai3). Bars represent mean ± SEM. ns, not significant.

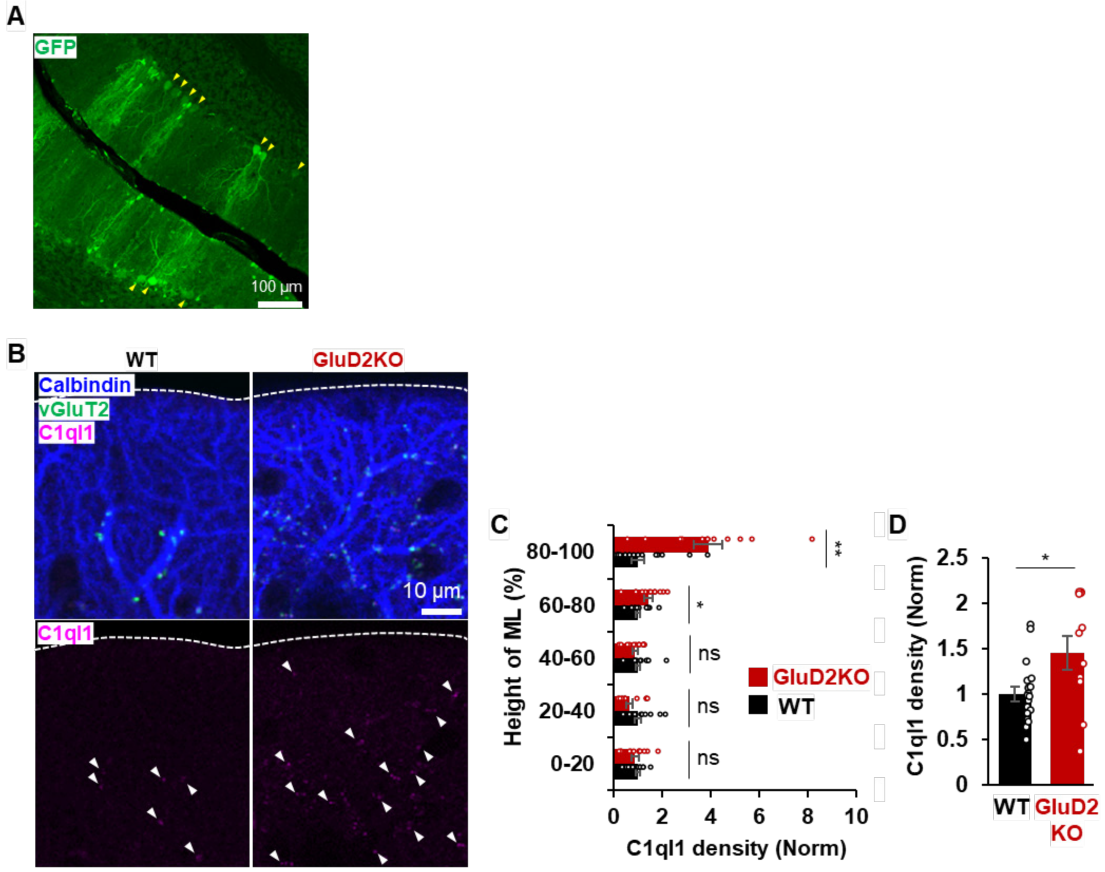

Supplementary Fig. 7, related to Fig. 5: Effect of conditional GluD2 knockout on endogenous C1ql1 levels in the adult cerebellum.

A Representative image of the lentivirus-based sparse expression of GFP-F2A-Cre in the cerebellum. Yellow arrowheads indicate infected PCs. **B** Representative images showing C1ql1 (magenta), calbindin (blue), and vGluT2 (green) in wild-type (WT) and Cre-mediated GluD2 knockout (KO) cerebellum at 2-3 months of age. White arrowheads indicate C1ql1 immunopositive puncta. **C** Histogram showing the mean C1ql1 density by their height in the molecular layer of WT and conditional GluD2 KO mice. C1ql1 density is normalized by WT. 0-20%, $p = 0.4757$; 20-40%, $p = 0.0577$; 40-60%, $p = 0.3847$; 60-80%, $p = 0.0282$; 80-100%, $p = 0.0003513$. Two-tailed Welch's t-test. $n = 19$ areas (WT) and $n = 12$ areas (GluD2 KO) from 2 mice for each group. **D** Histogram showing the mean C1ql1 density in the molecular layer of WT and GluD2 KO mice. C1ql1 density is normalized by WT. $p = 0.0342$. Two-tailed Welch's t-test. $n = 19$ areas (WT) and $n = 12$ areas (GluD2 KO) from 2 mice for each group.

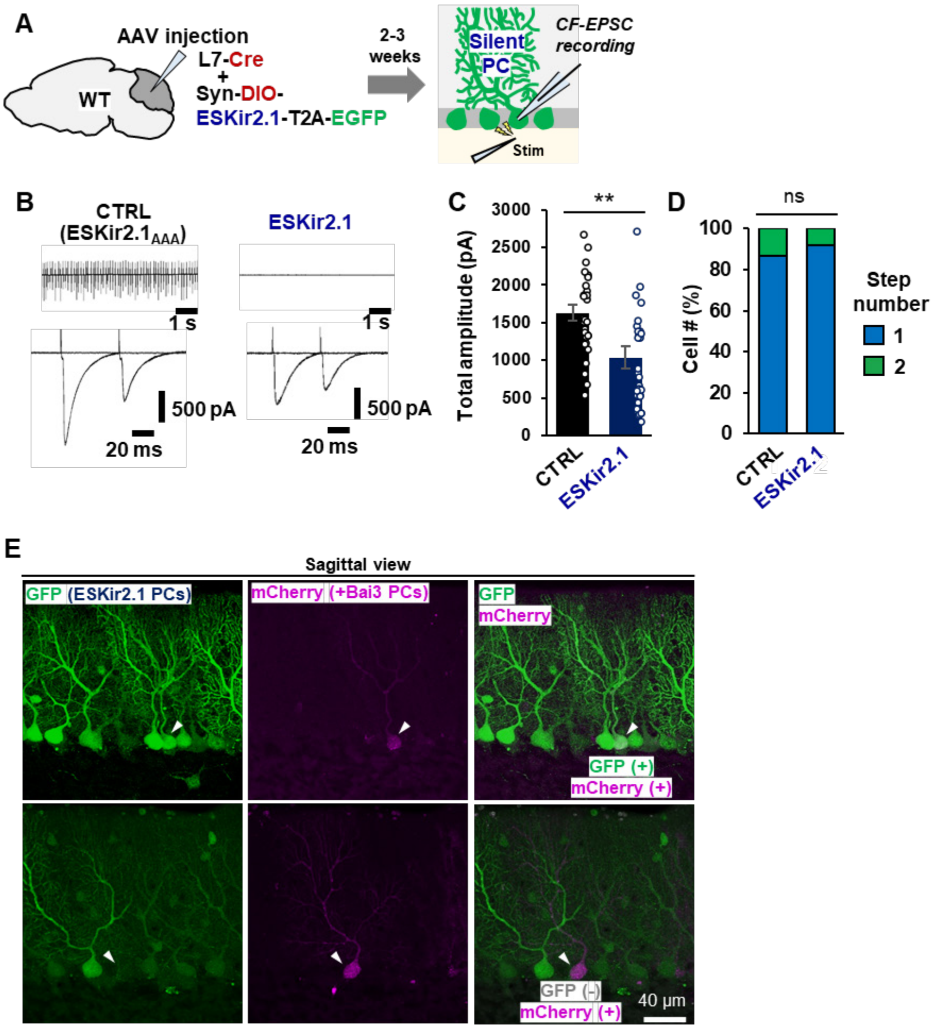

Supplementary Fig. 8, related to Fig. 6: AAV-mediated expression of ESKir2.1 and lentivirus-mediated expression of Bai3 in wild-type PCs.

A Diagram showing PC-specific expression of ESKir2.1 to silence PC activities. **B** Representative action potentials by loose-patch recordings (top traces) and CF-EPSCs by whole-cell patch-clamp recordings (bottom traces) from PCs expressing the indicated constructs. **C** Silencing PCs reduced total CF-EPSC amplitude. The graph shows the sum of peak amplitudes of single CF-EPSCs or multiple CF-EPSCs. $p = 0.0018$, two-tailed Welch's t-test. $n = 31$ cells from 6 mice (CTRL), $n = 32$ cells from 6 mice (ESKir2.1). **D** Silencing PCs did not affect the percentage of the number of CFs innervating single PCs. The number of EPSCs evoked by distinct CF activation thresholds (the number of steps) is shown. $p = 0.4750$, Mann–Whitney U test, $n = 46$ cells from 7 mice (CTRL), $n = 37$ cells from 7 mice (ESKir2.1). **E** Representative images showing Bai3-overexpressing PCs by lentivirus (mCherry-positive, magenta) and ESKir2.1 expressing PCs by AAV (GFP-positive, green). Bai3-overexpressing PCs are usually surrounded by many ESKir2.1-expressing PCs.

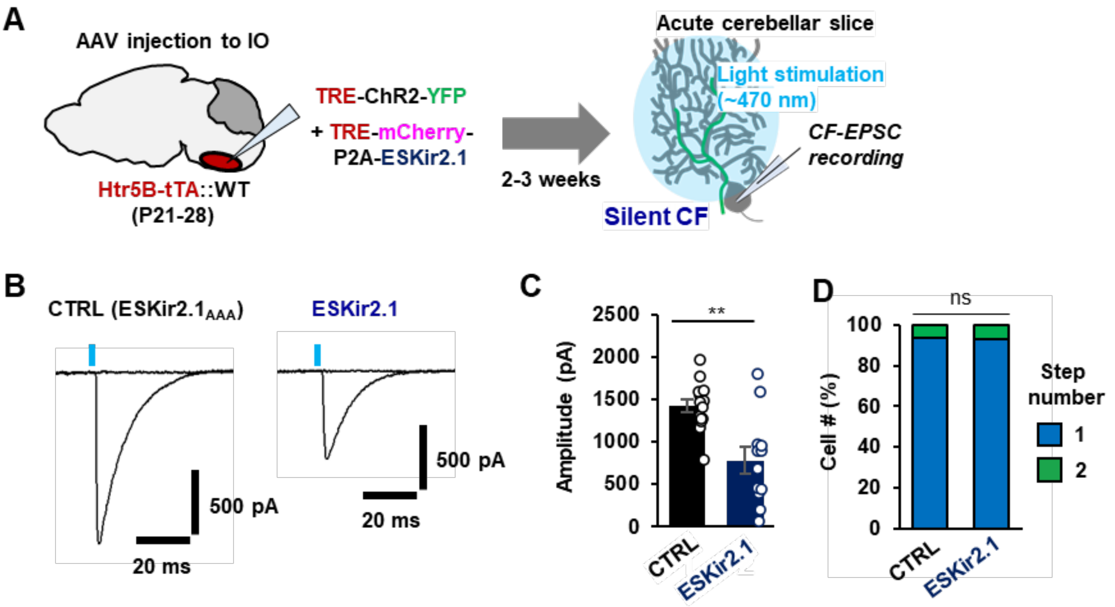

Supplementary Fig. 9, related to Fig. 7: The effect of CF silencing on CF-EPSC.

A Experimental scheme. **B** Representative traces of light-evoked EPSCs (blue lines). **C** Total CF-EPSC amplitude. The graph shows the sum of the peak amplitudes of single CF-EPSCs or multiple CF-EPSCs. p = 0.0015, Two-tailed Welch's t-test. n = 14 (CTRL), n = 12 (ESKir2.1). **D** The percentage of the number of CFs innervating single PCs. p = 0.9234, Mann–Whitney U test; n = 16 cells from 4 mice (CTRL); n = 14 cells from 4 mice (ESKir2.1).

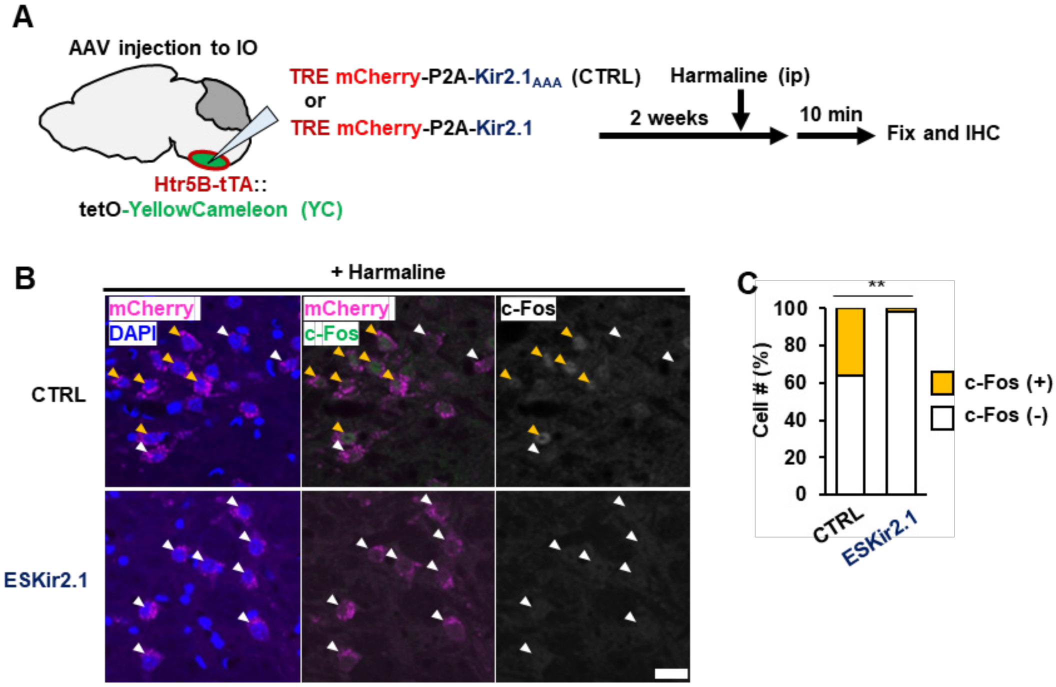

Supplementary Fig. 10, related to Fig. 7: Neuronal activity is suppressed by the expression of ESKir2.1 in the inferior olive.

A Experimental scheme to investigate the suppressive effect of ESKir2.1 on the harmaline-induced increase in c-Fos expression in the IONs. **B** Representative immunohistochemical images of the IONs. c-Fos immunoreactivity (yellow arrowheads) was detected in neurons expressing $ESKir_{AAA}$ (CTRL, top panels, white arrowheads) but not in neurons expressing ESKir2.1 (bottom panels, white arrowheads). Scale bar, 20 mm. **C** The percentage of cells showing c-Fos immunoreactivity. $p = 9.326 \times 10^{-15}$, Mann–Whitney U test. n = 166 cells from 3 mice (CTRL), n = 157 cells from 3 mice (ESKir2.1). $^{**}p < 0.01$.

Supplementary Method

Harmaline injection

Harmaline solution (3 mg/mL) was prepared by using 0.2 N HCl/PBS. AAV-injected mice were treated with harmaline solution (30 mg/kg) by intraperitoneal injection. Ten min after harmaline injection, mice were anesthetized by intraperitoneal injection of 2% avertin and fixed by perfusion with 4% PFA/0.1M sodium phosphate buffer. Immunohistochemical analysis was performed after fixation.

Immunoblotting

The cerebellum was dissected and homogenized in 320 mM sucrose in buffer A (10 mM Tris-HCl and 1 mM EDTA, pH7.5) and centrifuged at 800 g for 5 min. The supernatant was centrifuged at 12,000 *g* for 20 min, and the resulting pellet (P2 fraction) was resuspended in 1% Triton X-100 in PBS for 1 h at 4 °C. Followed by centrifugation at 100,000 *g* for 30 min, the postsynaptic density (PSD) fraction was obtained. Samples were diluted in Laemmli buffer and incubated with 2% 2-mercaptoethanol for 20 min at 70 °C. Samples were subjected to gradient gel SDS-PAGE (Fujifilm Wako Chemicals) and blotted onto membranes (Immobilon-P PVDF membrane, Merck Millipore). The membrane was blocked with Tris-HCl buffer (0.1% Tween-20, 50 mM Tris-HCl pH 7.6, 150 mM NaCl) containing 5% skimmed milk (CP-Meiji) and subjected to anti-Bai3 antibody (rabbit) for 1 h and HRP-conjugated secondary antibody for 1 h. Chemiluminescence was generated by using the Immobilon Western Chemiluminescent HRP Substrate (Millipore) detection kit and detected by iBright systems (Thermo Fisher Scientific).
